# Burden of injury along the development spectrum: associations between the Socio-demographic Index and disability-adjusted life year estimates from the Global Burden of Disease Study 2017

**DOI:** 10.1136/injuryprev-2019-043296

**Published:** 2020-01-08

**Authors:** Juanita A Haagsma, Spencer L James, Chris D Castle, Zachary V Dingels, Jack T Fox, Erin B Hamilton, Zichen Liu, Lydia R Lucchesi, Nicholas L S Roberts, Dillon O Sylte, Oladimeji M Adebayo, Alireza Ahmadi, Muktar Beshir Ahmed, Miloud Taki Eddine Aichour, Fares Alahdab, Suliman A Alghnam, Syed Mohamed Aljunid, Rajaa M Al-Raddadi, Ubai Alsharif, Khalid Altirkawi, Mina Anjomshoa, Carl Abelardo T Antonio, Seth Christopher Yaw Appiah, Olatunde Aremu, Amit Arora, Hamid Asayesh, Reza Assadi, Ashish Awasthi, Beatriz Paulina Ayala Quintanilla, Shivanthi Balalla, Amrit Banstola, Suzanne Lyn Barker-Collo, Till Winfried Bärnighausen, Shahrzad Bazargan-Hejazi, Neeraj Bedi, Masoud Behzadifar, Meysam Behzadifar, Corina Benjet, Derrick A Bennett, Isabela M Bensenor, Soumyadeep Bhaumik, Zulfiqar A Bhutta, Ali Bijani, Guilherme Borges, Rohan Borschmann, Dipan Bose, Soufiane Boufous, Alexandra Brazinova, Julio Cesar Campuzano Rincon, Rosario Cárdenas, Juan J Carrero, Félix Carvalho, Carlos A Castañeda-Orjuela, Ferrán Catalá-López, Jee-Young J Choi, Devasahayam J Christopher, Christopher Stephen Crowe, Koustuv Dalal, Ahmad Daryani, Dragos Virgil Davitoiu, Louisa Degenhardt, Diego De Leo, Jan-Walter De Neve, Kebede Deribe, Getenet Ayalew Dessie, Gabrielle Aline deVeber, Samath Dhamminda Dharmaratne, Linh Phuong Doan, Kate A Dolan, Tim Robert Driscoll, Manisha Dubey, Ziad El-Khatib, Christian Lycke Ellingsen, Maysaa El Sayed Zaki, Aman Yesuf Endries, Sharareh Eskandarieh, Andre Faro, Seyed-Mohammad Fereshtehnejad, Eduarda Fernandes, Irina Filip, Florian Fischer, Richard Charles Franklin, Takeshi Fukumoto, Kebede Embaye Gezae, Tiffany K Gill, Alessandra C Goulart, Ayman Grada, Yuming Guo, Rahul Gupta, Hassan Haghparast Bidgoli, Arvin Haj-Mirzaian, Arya Haj-Mirzaian, Randah R Hamadeh, Samer Hamidi, Josep Maria Haro, Hadi Hassankhani, Hamid Yimam Hassen, Rasmus Havmoeller, Delia Hendrie, Andualem Henok, Martha Híjar, Michael K Hole, Enayatollah Homaie Rad, Naznin Hossain, Sorin Hostiuc, Guoqing Hu, Ehimario U Igumbor, Olayinka Stephen Ilesanmi, Seyed Sina Naghibi Irvani, Sheikh Mohammed Shariful Islam, Rebecca Q Ivers, Kathryn H Jacobsen, Nader Jahanmehr, Mihajlo Jakovljevic, Achala Upendra Jayatilleke, Ravi Prakash Jha, Jost B Jonas, Zahra Jorjoran Shushtari, Jacek Jerzy Jozwiak, Mikk Jürisson, Ali Kabir, Rizwan Kalani, Amir Kasaeian, Abraham Getachew Kelbore, Andre Pascal Kengne, Yousef Saleh Khader, Morteza Abdullatif Khafaie, Nauman Khalid, Ejaz Ahmad Khan, Abdullah T Khoja, Aliasghar A Kiadaliri, Young-Eun Kim, Daniel Kim, Adnan Kisa, Ai Koyanagi, Barthelemy Kuate Defo, Burcu Kucuk Bicer, Manasi Kumar, Ratilal Lalloo, Hilton Lam, Faris Hasan Lami, Van C Lansingh, Janet L Leasher, Shanshan Li, Shai Linn, Raimundas Lunevicius, Flavia R Machado, Hassan Magdy Abd El Razek, Muhammed Magdy Abd El Razek, Narayan Bahadur Mahotra, Marek Majdan, Azeem Majeed, Reza Malekzadeh, Manzoor Ahmad Malik, Deborah Carvalho Malta, Ana-Laura Manda, Mohammad Ali Mansournia, Benjamin Ballard Massenburg, Pallab K Maulik, Hailemariam Abiy Alemu Meheretu, Man Mohan Mehndiratta, Addisu Melese, Walter Mendoza, Melkamu Merid Mengesha, Tuomo J Meretoja, Atte Meretoja, Tomislav Mestrovic, Tomasz Miazgowski, Ted R Miller, GK Mini, Erkin M Mirrakhimov, Babak Moazen, Naser Mohammad Gholi Mezerji, Roghayeh Mohammadibakhsh, Shafiu Mohammed, Mariam Molokhia, Lorenzo Monasta, Stefania Mondello, Pablo A Montero-Zamora, Yoshan Moodley, Mahmood Moosazadeh, Ghobad Moradi, Maziar Moradi-Lakeh, Lidia Morawska, Ilais Moreno Velásquez, Shane Douglas Morrison, Marilita M Moschos, Seyyed Meysam Mousavi, Srinivas Murthy, Kamarul Imran Musa, Gurudatta Naik, Farid Najafi, Vinay Nangia, Bruno Ramos Nascimento, Duduzile Edith Ndwandwe, Ionut Negoi, Trang Huyen Nguyen, Son Hoang Nguyen, Long Hoang Nguyen, Huong Lan Thi Nguyen, Dina Nur Anggraini Ningrum, Yirga Legesse Nirayo, Richard Ofori-Asenso, Felix Akpojene Ogbo, In-Hwan Oh, Olanrewaju Oladimeji, Andrew T Olagunju, Tinuke O Olagunju, Pedro R Olivares, Heather M Orpana, Stanislav S Otstavnov, Mahesh P A, Smita Pakhale, Eun-Kee Park, George C Patton, Konrad Pesudovs, Michael R Phillips, Suzanne Polinder, Swayam Prakash, Amir Radfar, Anwar Rafay, Alireza Rafiei, Siavash Rahimi, Vafa Rahimi-Movaghar, Muhammad Aziz Rahman, Rajesh Kumar Rai, Kiana Ramezanzadeh, Salman Rawaf, David Laith Rawaf, Andre M N Renzaho, Serge Resnikoff, Shahab Rezaeian, Leonardo Roever, Luca Ronfani, Gholamreza Roshandel, Yogesh Damodar Sabde, Basema Saddik, Payman Salamati, Yahya Salimi, Inbal Salz, Abdallah M Samy, Juan Sanabria, Lidia Sanchez Riera, Milena M Santric Milicevic, Maheswar Satpathy, Monika Sawhney, Susan M Sawyer, Sonia Saxena, Mete Saylan, Ione J C Schneider, David C Schwebel, Soraya Seedat, Sadaf G Sepanlou, Masood Ali Shaikh, Mehran Shams-Beyranvand, Morteza Shamsizadeh, Mahdi Sharif-Alhoseini, Aziz Sheikh, Jiabin Shen, Mika Shigematsu, Rahman Shiri, Ivy Shiue, João Pedro Silva, Jasvinder A Singh, Dhirendra Narain Sinha, Adauto Martins Soares Filho, Joan B Soriano, Sergey Soshnikov, Ireneous N Soyiri, Vladimir I Starodubov, Dan J Stein, Mark A Stokes, Mu'awiyyah Babale Sufiyan, Jacob E Sunshine, Bryan L Sykes, Rafael Tabarés-Seisdedos, Karen M Tabb, Arash Tehrani-Banihashemi, Gizachew Assefa Tessema, Jarnail Singh Thakur, Khanh Bao Tran, Bach Xuan Tran, Lorainne Tudor Car, Olalekan A Uthman, Benjamin S Chudi Uzochukwu, Pascual R Valdez, Elena Varavikova, Ana Maria Nogales Vasconcelos, Narayanaswamy Venketasubramanian, Francesco S Violante, Vasily Vlassov, Yasir Waheed, Yuan-Pang Wang, Tissa Wijeratne, Andrea Sylvia Winkler, Priyanka Yadav, Yuichiro Yano, Muluken Azage Yenesew, Paul Yip, Engida Yisma, Naohiro Yonemoto, Mustafa Z Younis, Chuanhua Yu, Shamsa Zafar, Zoubida Zaidi, Sojib Bin Zaman, Mohammad Zamani, Yong Zhao, Sanjay Zodpey, Simon I Hay, Alan D Lopez, Ali H Mokdad, Theo Vos

**Affiliations:** 1 Department of Public Health, Erasmus University Medical Center, Rotterdam, The Netherlands; 2 Institute for Health Metrics and Evaluation, University of Washington, Seattle, Washington, USA; 3 Department of Medicine, University College Hospital Ibadan, Ibadan, Nigeria; 4 Department of Anesthesiology, Kermanshah University of Medical Sciences, Kermanshah, Iran; 5 Department of Epidemiology, Jimma University, Jimma, Ethiopia; 6 Higher National School of Veterinary Medicine, Algiers, Algeria; 7 Evidence Based Practice Center, Mayo Clinic Foundation for Medical Education and Research, Rochester, Minnesota, USA; 8 Department of Population Health Research, King Abdullah International Medical Research Center, Riyadh, Saudi Arabia; 9 Department of Health Policy and Management, Kuwait University, Safat, Kuwait; 10 International Centre for Casemix and Clinical Coding, National University of Malaysia, Bandar Tun Razak, Malaysia; 11 Department of Family and Community Medicine, King Abdulaziz University, Jeddah, Saudi Arabia; 12 Department of Oral and Maxillofacial Surgery, University Hospital Knappschaftskrankenhaus Bochum, Bochum, Germany; 13 King Saud University, Riyadh, Saudi Arabia; 14 Social Determinants of Health Research Center, Rafsanjan University of Medical Sciences, Rafsanjan, Iran; 15 Department of Health Policy and Administration, University of the Philippines Manila, Manila, Philippines; 16 Department of Applied Social Sciences, Hong Kong Polytechnic University, Hong Kong, China; 17 Department of Sociology and Social Work, Kwame Nkrumah University of Science and Technology, Kumasi, Ghana; 18 Center for International Health, Ludwig Maximilians University, Munich, Germany; 19 School of Health Sciences, Birmingham City University, Birmingham, UK; 20 School of Science and Health, Western Sydney University, Sydney, New South Wales, Australia; 21 Oral Health Services, Sydney Local Health District, Sydney, New South Wales, Australia; 22 Qom University of Medical Sciences, Qom, Iran; 23 Education Development Center, Mashhad University of Medical Sciences, Mashhad, Iran; 24 Indian Institute of Public Health, Gandhinagar, India; 25 The Judith Lumley Centre, La Trobe University, Melbourne, Victoria, Australia; 26 General Office for Research and Technological Transfer, Peruvian National Institute of Health, Lima, Peru; 27 School of Public Health, Auckland University of Technology, Auckland, New Zealand; 28 Department of Research, Public Health Perspective Nepal, Pokhara-Lekhnath Metropolitan City, Nepal; 29 School of Psychology, University of Auckland, Auckland, New Zealand; 30 Heidelberg Institute of Global Health (HIGH), Heidelberg University, Heidelberg, Germany; 31 T.H. Chan School of Public Health, Harvard University, Boston, Massachusetts, USA; 32 Department of Psychiatry, Charles R. Drew University of Medicine and Science, Los Angeles, California, USA; 33 Department of Psychiatry and Biobehavioral Sciences, David Geffen School of Medicine, University of California Los Angeles, Los Angeles, California, USA; 34 Department of Community Medicine, Gandhi Medical College Bhopal, Bhopal, India; 35 Social Determinants of Health Research Center, Lorestan University of Medical Sciences, Khorramabad, Iran; 36 Department of Epidemiology and Biostatistics, Lorestan University of Medical Sciences, Khorramabad, Iran; 37 Department of Epidemiology and Psychosocial Research, Ramón de la Fuente Muñiz National Institute of Psychiatry, Mexico City, Mexico; 38 Nuffield Department of Population Health, University of Oxford, Oxford, UK; 39 Department of Internal Medicine, University of São Paulo, São Paulo, Brazil; 40 The George Institute for Global Health, New Delhi, India; 41 Centre for Global Child Health, The Hospital for Sick Children, Toronto, Ontario, Canada; 42 Centre of Excellence in Women and Child Health, Aga Khan University, Karachi, Pakistan; 43 Social Determinants of Health Research Center, Babol University of Medical Sciences, Babol, Iran; 44 Centre for Adolescent Health, Murdoch Childrens Research Institute, Melbourne, Victoria, Australia; 45 School of Population and Global Health, University of Melbourne, Melbourne, Victoria, Australia; 46 Transport & Digital Development, World Bank, Washington, District of Columbia, USA; 47 Transport and Road Safety (TARS) Research Department, University of New South Wales, Sydney, New South Wales, Australia; 48 Institute of Epidemiology, Comenius University, Bratislava, Slovakia; 49 National Institute of Public Health, Cuernavaca, Mexico; 50 School of Medicine, University of the Valley of Cuernavaca, Cuernavaca, Mexico; 51 Department of Population and Health, Metropolitan Autonomous University, Mexico City, Mexico; 52 Department of Medical Epidemiology and Biostatistics, Karolinska Institutet, Stockholm, Sweden; 53 UCIBIO, University of Porto, Porto, Portugal; 54 Colombian National Health Observatory, National Institute of Health, Bogota, Colombia; 55 Epidemiology and Public Health Evaluation Group, National University of Colombia, Bogota, Colombia; 56 National School of Public Health, Carlos III Health Institute, Madrid, Spain; 57 Clinical Epidemiology Program, Ottawa Hospital Research Institute, Ottawa, Ontario, Canada; 58 Department of Biochemistry and Biomedical Science, Seoul National University Hospital, Seoul, South Korea; 59 Department of Pulmonary Medicine, Christian Medical College and Hospital (CMC), Vellore, India; 60 Division of Plastic Surgery, University of Washington, Seattle, Washington, USA; 61 Institute of Public Health Kalyani, Kalyani, India; 62 School of Health Science, Orebro University, Orebro, Sweden; 63 Toxoplasmosis Research Center, Mazandaran University of Medical Sciences, Sari, Iran; 64 Department of General Surgery, Carol Davila University of Medicine and Pharmacy, Bucharest, Romania; 65 Department of Surgery, Clinical Emergency Hospital Sf. Pantelimon, Bucharest, Romania; 66 National Drug and Alcohol Research Centre, University of New South Wales, Sydney, New South Wales, Australia; 67 Australian Institute for Suicide Research and Prevention, Griffith University, Mount Gravatt, Queensland, Australia; 68 Department of Global Health and Infection, Brighton and Sussex Medical School, Brighton, UK; 69 School of Public Health, Addis Ababa University, Addis Ababa, Ethiopia; 70 Department of Nursing, Debre Markos University, Debre Markos, Ethiopia; 71 Centre for Global Child Health, The Hospital for Sick Children, University of Toronto, Toronto, Ontario, Canada; 72 Department of Community Medicine, University of Peradeniya, Peradeniya, Sri Lanka; 73 Center of Excellence in Health Service Management, Nguyen Tat Thanh University, Ho Chi Minh, Vietnam; 74 University of New South Wales, Sydney, New South Wales, Australia; 75 Sydney School of Public Health, University of Sydney, Sydney, New South Wales, Australia; 76 United Nations World Food Programme, New Delhi, India; 77 Department of Public Health Sciences, Karolinska Institutet, Stockholm, Sweden; 78 World Health Programme, Université du Québec en Abitibi-Témiscamingue, Rouyn-Noranda, Quebec, Canada; 79 Department of Pathology, Stavanger University Hospital, Stavanger, Norway; 80 Department of Clinical Pathology, Mansoura University, Mansoura, Egypt; 81 Public Health Department, Saint Paul’s Hospital Millennium Medical College, Addis Ababa, Ethiopia; 82 Multiple Sclerosis Research Center, Tehran University of Medical Sciences, Tehran, Iran; 83 Department of Psychology, Federal University of Sergipe, Sao Cristovao, Brazil; 84 Department of Neurobiology, Care Sciences and Society, Karolinska Institutet, Stockholm, Sweden; 85 Division of Neurology, University of Ottawa, Ottawa, Ontario, Canada; 86 REQUIMTE/LAQV, University of Porto, Porto, Portugal; 87 Psychiatry Department, Kaiser Permanente, Fontana, California, USA; 88 School of Health Sciences, A.T. Still University, Mesa, Arizona, USA; 89 Department of Population Medicine and Health Services Research, Bielefeld University, Bielefeld, Germany; 90 College of Public Health, Medical and Veterinary Science, James Cook University, Douglas, Queensland, Australia; 91 Gene Expression & Regulation Program, The Wistar Institute, Philadelphia, Pennsylvania, USA; 92 Department of Dermatology, Kobe University, Kobe, Japan; 93 Department of Biostatistics, Mekelle University, Mekelle, Ethiopia; 94 Adelaide Medical School, University of Adelaide, Adelaide, South Australia, Australia; 95 Center for Clinical and Epidemiological Research, University of São Paulo, Sao Paulo, Brazil; 96 Internal Medicine Department, University Hospital, University of São Paulo, Sao Paulo, Brazil; 97 School of Medicine, Boston University, Boston, Massachusetts, USA; 98 School of Public Health and Preventive Medicine, Monash University, Melbourne, Victoria, Australia; 99 Department of Epidemiology and Biostatistics, Zhengzhou University, Zhengzhou, China; 100 March of Dimes, Arlington, Virginia, USA; 101 School of Public Health, West Virginia University, Morgantown, West Virginia, USA; 102 Institute for Global Health, University College London, London, UK; 103 Department of Pharmacology, Tehran University of Medical Sciences, Tehran, Iran; 104 Obesity Research Center, Shahid Beheshti University of Medical Sciences, Tehran, Iran; 105 Department of Radiology, Johns Hopkins University, Baltimore, Maryland, USA; 106 Department of Family and Community Medicine, Arabian Gulf University, Manama, Bahrain; 107 School of Health and Environmental Studies, Hamdan Bin Mohammed Smart University, Dubai, United Arab Emirates; 108 Biomedical Research Networking Center for Mental Health Network (CiberSAM), Madrid, Spain; 109 Research and Development Unit, San Juan de Dios Sanitary Park, Sant Boi de Llobregat, Spain; 110 School of Nursing and Midwifery, Tabriz University of Medical Sciences, Tabriz, Iran; 111 Independent Consultant, Tabriz, Iran; 112 Department of Public Health, Mizan-Tepi University, Teppi, Ethiopia; 113 Unit of Epidemiology and Social Medicine, University Hospital Antwerp, Wilrijk, Belgium; 114 Clinical Sciences, Karolinska University Hospital, Stockholm, Sweden; 115 School of Public Health, Curtin University, Perth, Western Australia, Australia; 116 Research Coordination, AC Environments Foundation, Cuernavaca, Mexico; 117 CISS, National Institute of Public Health, Cuernavaca, Mexico; 118 Department of Pediatrics, Dell Medical School, University of Texas Austin, Austin, Texas, USA; 119 Guilan Road Trauma Research Center, Guilan University of Medical Sciences, Rasht, Iran; 120 Social Determinants of Health Research Center, Guilan University of Medical Sciences, Rasht, Iran; 121 Department of Pharmacology and Therapeutics, Dhaka Medical College, Dhaka University, Dhaka, Bangladesh; 122 Department of Pharmacology, Bangladesh Industrial Gases Limited, Tangail, Bangladesh; 123 Faculty of Dentistry, Department of Legal Medicine and Bioethics, Carol Davila University of Medicine and Pharmacy, Bucharest, Romania; 124 Clinical Legal Medicine Department, National Institute of Legal Medicine Mina Minovici, Bucharest, Romania; 125 Department of Epidemiology and Health Statistics, Central South University, Changsha, China; 126 School of Public Health, University of the Western Cape, Bellville, Cape Town, South Africa; 127 Department of Public Health, Walter Sisulu University, Mthatha, South Africa; 128 Department of Community Medicine, University of Ibadan, Ibadan, Nigeria; 129 Research Institute for Endocrine Sciences, Shahid Beheshti University of Medical Sciences, Tehran, Iran; 130 Institute for Physical Activity and Nutrition, Deakin University, Burwood, Victoria, Australia; 131 Sydney Medical School, University of Sydney, Sydney, New South Wales, Australia; 132 Injury Division, The George Institute for Global Health, Newtown, New South Wales, Australia; 133 Department of Global and Community Health, George Mason University, Fairfax, Virginia, USA; 134 School of Management and Medical Education, Shahid Beheshti University of Medical Sciences, Tehran, Iran; 135 Safety Promotion and Injury Prevention Research Center, Shahid Beheshti University of Medical Sciences, Tehran, Iran; 136 Department for Health Care and Public Health, I.M. Sechenov First Moscow State Medical University, Moscow, Russia; 137 Institute of Medicine, University of Colombo, Colombo, Sri Lanka; 138 Faculty of Graduate Studies, University of Colombo, Colombo, Sri Lanka; 139 Department of Community Medicine, Banaras Hindu University, Varanasi, India; 140 Department of Ophthalmology, Heidelberg University, Mannheim, Germany; 141 Beijing Institute of Ophthalmology, Beijing Tongren Hospital, Beijing, China; 142 Social Determinants of Health Research Center, University of Social Welfare and Rehabilitation Sciences, Tehran, Iran; 143 Department of Family Medicine and Public Health, University of Opole, Opole, Poland; 144 Institute of Family Medicine and Public Health, University of Tartu, Tartu, Estonia; 145 Minimally Invasive Surgery Research Center, Iran University of Medical Sciences, Tehran, Iran; 146 Department of Neurology, University of Washington, Seattle, Washington, USA; 147 Hematology-Oncology and Stem Cell Transplantation Research Center, Tehran University of Medical Sciences, Tehran, Iran; 148 Pars Advanced and Minimally Invasive Medical Manners Research Center, Iran University of Medical Sciences, Tehran, Iran; 149 Department of Dermatology, Wolaita Sodo University, Wolaita Sodo, Ethiopia; 150 Non-communicable Diseases Research Unit, Medical Research Council South Africa, Cape Town, South Africa; 151 Department of Medicine, University of Cape Town, Cape Town, South Africa; 152 Department of Public Health and Community Medicine, Jordan University of Science and Technology, Ramtha, Jordan; 153 Social Determinants of Health Research Center, Ahvaz Jundishapur University of Medical Sciences, Ahvaz, Iran; 154 School of Food and Agricultural Sciences, University of Management and Technology, Lahore, Pakistan; 155 Epidemiology and Biostatistics Department, Health Services Academy, Islamabad, Pakistan; 156 Department of Public Health, Imam Muhammad Ibn Saud Islamic University, Riyadh, Saudi Arabia; 157 Department of Health Policy and Management, Johns Hopkins University, Baltimore, Maryland, USA; 158 Clinical Epidemiology Unit, Lund University, Lund, Sweden; 159 Department of Preventive Medicine, Korea University, Seoul, South Korea; 160 Department of Health Sciences, Northeastern University, Boston, Massachusetts, USA; 161 School of Health Sciences, Kristiania University College, Oslo, Norway; 162 CIBERSAM, San Juan de Dios Sanitary Park, Sant Boi de Llobregat, Spain; 163 Catalan Institution for Research and Advanced Studies (ICREA), Barcelona, Spain; 164 Department of Demography, University of Montreal, Montreal, Quebec, Canada; 165 Department of Social and Preventive Medicine, University of Montreal, Montreal, Quebec, Canada; 166 Department of Public Health, Yuksek Ihtisas University, Ankara, Turkey; 167 Department of Public Health, Hacettepe University, Ankara, Turkey; 168 Department of Psychiatry, University of Nairobi, Nairobi, Kenya; 169 Division of Psychology and Language Sciences, University College London, London, UK; 170 School of Dentistry, The University of Queensland, Brisbane, Queensland, Australia; 171 Institute of Health Policy and Development Studies, National Institutes of Health, Manila, Philippines; 172 Department of Community and Family Medicine, University of Baghdad, Baghdad, Iraq; 173 HelpMeSee, New York City, New York, USA; 174 International Relations Department, Mexican Institute of Ophthalmology, Queretaro, Mexico; 175 College of Optometry, Nova Southeastern University, Fort Lauderdale, Florida, USA; 176 School of Public Health, University of Haifa, Haifa, Israel; 177 Department of General Surgery, Aintree University Hospital National Health Service (NHS) Foundation Trust, Liverpool, UK; 178 Department of Surgery, University of Liverpool, Liverpool, UK; 179 Anesthesiology, Pain and Intensive Care Department, Federal University of São Paulo, Sao Paulo, Brazil; 180 Radiology Department, Mansoura Faculty of Medicine, Mansoura, Egypt; 181 Ophthalmology Department, Aswan Faculty of Medicine, Aswan, Egypt; 182 Institute of Medicine, Tribhuvan University, Kathmandu, Nepal; 183 Department of Public Health, Trnava University, Trnava, Slovakia; 184 Department of Primary Care and Public Health, Imperial College London, London, UK; 185 Digestive Diseases Research Institute, Tehran University of Medical Sciences, Tehran, Iran; 186 Non-communicable Diseases Research Center, Shiraz University of Medical Sciences, Shiraz, Iran; 187 Department of Humanities and Social Sciences, Indian Institute of Technology, Roorkee, Haridwar, India; 188 Department of Development Studies, International Institute for Population Sciences, Mumbai, India; 189 Department of Maternal and Child Nursing and Public Health, Federal University of Minas Gerais, Belo Horizonte, Brazil; 190 Surgery Department, Emergency University Hospital Bucharest, Bucharest, Romania; 191 Department of Epidemiology and Biostatistics, Tehran University of Medical Sciences, Tehran, Iran; 192 Research Department, The George Institute for Global Health, New Delhi, India; 193 School of Medicine, University of New South Wales, Sydney, New South Wales, Australia; 194 School of Public Health, Bahir Dar University, Bahir Dar, Ethiopia; 195 Neurology Department, Janakpuri Super Specialty Hospital Society, New Delhi, India; 196 Neurology Department, Govind Ballabh Institute of Medical Education and Research, New Delhi, India; 197 Department of Medical Laboratory Sciences, Bahir Dar University, Bahir Dar, Ethiopia; 198 Peru Country Office, United Nations Population Fund (UNFPA), Lima, Peru; 199 Department of Epidemiology and Biostatistics, Haramaya University, Harar, Ethiopia; 200 Breast Surgery Unit, Helsinki University Hospital, Helsinki, Finland; 201 University of Helsinki, Helsinki, Finland; 202 Neurocenter, Helsinki University Hospital, Helsinki, Finland; 203 School of Health Sciences, University of Melbourne, Melbourne, Victoria, Australia; 204 Clinical Microbiology and Parasitology Unit, Zora Profozic Polyclinic, Zagreb, Croatia; 205 University Centre Varazdin, University North, Varazdin, Croatia; 206 Department of Propedeutics of Internal Diseases & Arterial Hypertension, Pomeranian Medical University, Szczecin, Poland; 207 Pacific Institute for Research & Evaluation, Calverton, Maryland, USA; 208 Achutha Menon Centre for Health Science Studies, Sree Chitra Tirunal Institute for Medical Sciences and Technology, Trivandrum, India; 209 Global Institute of Public Health (GIPH), Ananthapuri Hospitals and Research Centre, Trivandrum, India; 210 Faculty of Internal Medicine, Kyrgyz State Medical Academy, Bishkek, Kyrgyzstan; 211 Department of Atherosclerosis and Coronary Heart Disease, National Center of Cardiology and Internal Disease, Bishkek, Kyrgyzstan; 212 Institute of Addiction Research (ISFF), Frankfurt University of Applied Sciences, Frankfurt, Germany; 213 Department of Biostatistics, Hamadan University of Medical Sciences, Hamadan, Iran; 214 Hamadan University of Medical Sciences, Hamadan, Iran; 215 Health Systems and Policy Research Unit, Ahmadu Bello University, Zaria, Nigeria; 216 Faculty of Life Sciences and Medicine, King’s College London, London, UK; 217 Clinical Epidemiology and Public Health Research Unit, Burlo Garofolo Institute for Maternal and Child Health, Trieste, Italy; 218 Department of Biomedical and Dental Sciences and Morphofunctional Imaging, University of Messina, Messina, Italy; 219 Department of Neurology, Oasi Research Institute, Troina, Italy; 220 Department of Public Health Sciences, University of Miami, Miami, Florida, USA; 221 Center for Health Systems Research, National Institute of Public Health, Cuernavaca, Mexico; 222 Department of Public Health Medicine, University of KwaZulu-Natal, Durban, South Africa; 223 Health Sciences Research Center, Mazandaran University of Medical Sciences, Sari, Iran; 224 Social Determinants of Health Research Center, Kurdistan University of Medical Sciences, Sanandaj, Iran; 225 Department of Epidemiology and Biostatistics, Kurdistan University of Medical Sciences, Sanandaj, Iran; 226 Preventive Medicine and Public Health Research Center, Iran University of Medical Sciences, Tehran, Iran; 227 International Laboratory for Air Quality and Health, Queensland University of Technology, Brisbane, Queensland, Australia; 228 Gorgas Memorial Institute for Health Studies, Panama City, Panama; 229 Department of Surgery, University of Washington, Seattle, Washington, USA; 230 1st Department of Ophthalmology, University of Athens, Athens, Greece; 231 Biomedical Research Foundation, Academy of Athens, Athens, Greece; 232 Health Management Reserach Center, Baqiyatallah University of Medical Sciences, Tehran, Iran; 233 Department of Health Management and Economics, Tehran University of Medical Sciences, Tehran, Iran; 234 Department of Pediatrics, University of British Columbia, Vancouver, British Columbia, Canada; 235 School of Medical Sciences, Science University of Malaysia, Kubang Kerian, Malaysia; 236 Department of Epidemiology, University of Alabama at Birmingham, Birmingham, Alabama, USA; 237 Department of Epidemiology & Biostatistics, Kermanshah University of Medical Sciences, Kermanshah, Iran; 238 Suraj Eye Institute, Nagpur, India; 239 Hospital of the Federal University of Minas Gerais, Federal University of Minas Gerais, Belo Horizonte, Brazil; 240 Cochrane South Africa, South African Medical Research Council, Cape Town, South Africa; 241 General Surgery Department, Emergency Hospital of Bucharest, Bucharest, Romania; 242 Center of Excellence in Behavioral Medicine, Nguyen Tat Thanh University, Ho Chi Minh, Vietnam; 243 Institute for Global Health Innovations, Duy Tan University, Hanoi, Vietnam; 244 Public Health Department, Universitas Negeri Semarang, Kota Semarang, Indonesia; 245 Graduate Institute of Biomedical Informatics, Taipei Medical University, Taipei City, Taiwan; 246 Clinical Pharmacy Unit, Mekelle University, Mekelle, Ethiopia; 247 Centre of Cardiovascular Research and Education in Therapeutics, Monash University, Melbourne, Victoria, Australia; 248 Independent Consultant, Accra, Ghana; 249 Translational Health Research Institute, Western Sydney University, Penrith, New South Wales, Australia; 250 Department of Preventive Medicine, Kyung Hee University, Dongdaemun-gu, South Korea; 251 HAST, Human Sciences Research Council, Durban, South Africa; 252 School of Public Health, Faculty of Health Sciences, University of Namibia, Osakhati, Namibia; 253 Department of Psychiatry and Behavioural Neurosciences, McMaster University, Hamilton, ON, Canada; 254 Department of Psychiatry, University of Lagos, Lagos, Nigeria; 255 Department of Pathology and Molecular Medicine, McMaster University, Hamilton, Ontario, Canada; 256 Institute of Physical Activity and Health, Autonomous University of Chile, Talca, Chile; 257 Applied Research Division, Public Health Agency of Canada, Ottawa, Ontario, Canada; 258 School of Psychology, University of Ottawa, Ottawa, Ontario, Canada; 259 Analytical Center, Moscow Institute of Physics and Technology, Dolgoprudny, Russia; 260 Committee for the Comprehensive Assessment of Medical Devices and Information Technology, Health Technology Assessment Association, Moscow, Russia; 261 Department of Respiratory Medicine, Jagadguru Sri Shivarathreeswara Academy of Health Education and Research, Mysore, India; 262 Department of Medicine, Ottawa Hospital Research Institute, University of Ottawa, Ottawa, Ontario, Canada; 263 Department of Medical Humanities and Social Medicine, Kosin University, Busan, South Korea; 264 Department of Paediatrics, University of Melbourne, Melbourne, Victoria, Australia; 265 Population Health Department, Murdoch Childrens Research Institute, Melbourne, Victoria, Australia; 266 School of Optometry and Vision Science, University of New South Wales, Sydney, New South Wales, Australia; 267 Shanghai Mental Health Center, Shanghai Jiao Tong University, Shanghai, China; 268 Department of Psychiatry, Department of Epidemiology, Columbia University, New York City, New York, USA; 269 Department of Nephrology, Sanjay Gandhi Postgraduate Institute of Medical Sciences, Lucknow, India; 270 College of Medicine, University of Central Florida, Orlando, Florida, USA; 271 College of Graduate Health Sciences, A.T. Still University, Mesa, Arizona, USA; 272 Department of Epidemiology & Biostatistics, Contech School of Public Health, Lahore, Pakistan; 273 Department of Immunology, Mazandaran University of Medical Sciences, Sari, Iran; 274 Molecular and Cell Biology Research Center, Mazandaran University of Medical Sciences, Sari, Iran; 275 Faculty of Medicine, Mazandaran University of Medical Sciences, Sari, Iran; 276 Sina Trauma and Surgery Research Center, Tehran University of Medical Sciences, Tehran, Iran; 277 School of Nursing and Healthcare Professions, Federation University, Heidelberg, Victoria, Australia; 278 National Centre for Farmer Health, Deakin University, Waurn Ponds, Victoria, Australia; 279 Society for Health and Demographic Surveillance, Suri, India; 280 Department of Economics, University of Göttingen, Göttingen, Germany; 281 Department of Pharmacology, Shahid Beheshti University of Medical Sciences, Tehran, Iran; 282 Academic Public Health Department, Public Health England, London, UK; 283 WHO Collaborating Centre for Public Health Education and Training, Imperial College London, London, UK; 284 University College London Hospitals, London, UK; 285 School of Social Sciences and Psychology, Western Sydney University, Penrith, New South Wales, Australia; 286 Brien Holden Vision Institute, Sydney, New South Wales, Australia; 287 Organization for the Prevention of Blindness, Paris, France; 288 Kermanshah University of Medical Sciences, Kermanshah, Iran; 289 Department of Clinical Research, Federal University of Uberlândia, Uberlândia, Brazil; 290 Golestan Research Center of Gastroenterology and Hepatology, Golestan University of Medical Sciences, Gorgan, Iran; 291 National Institute for Research in Environmental Health, Indian Council of Medical Research, Bhopal, India; 292 College of Medicine, University of Sharjah, Sharjah, United Arab Emirates; 293 Social Development & Health Promotion Research Center, Kermanshah University of Medical Sciences, Kermanshah, Iran; 294 Health and Disability Intelligence Group, Ministry of Health, Wellington, New Zealand; 295 Department of Entomology, Ain Shams University, Cairo, Egypt; 296 Department of Surgery, Marshall University, Huntington, West Virginia, USA; 297 Department of Nutrition and Preventive Medicine, Case Western Reserve University, Cleveland, Ohio, USA; 298 Rheumatology Department, University Hospitals Bristol NHS Foundation Trust, Bristol, UK; 299 Institute of Bone and Joint Research, University of Sydney, Syndey, New South Wales, Australia; 300 Institute of Social Medicine, University of Belgrade, Belgrade, Serbia; 301 Centre-School of Public Health and Health Management, University of Belgrade, Belgrade, Serbia; 302 UGC Centre of Advanced Study in Psychology, Utkal University, Bhubaneswar, India; 303 Udyam-Global Association for Sustainable Development, Bhubaneswar, India; 304 Department of Public Health Sciences, University of North Carolina at Charlotte, Charlotte, North Carolina, USA; 305 School of Public Health, Imperial College London, London, UK; 306 Market Access Department, Bayer, Istanbul, Turkey; 307 School of Health Sciences, Federal University of Santa Catarina, Ararangua, Brazil; 308 Department of Psychology, University of Alabama at Birmingham, Birmingham, Alabama, USA; 309 Department of Psychiatry, Stellenbosch University, Cape Town, South Africa; 310 Independent Consultant, Karachi, Pakistan; 311 School of Medicine, Dezful University of Medical Sciences, Dezful, Iran; 312 School of Medicine, Alborz University of Medical Sciences, Karaj, Iran; 313 Chronic Diseases (Home Care) Research Center, Hamadan University of Medical Sciences, Hamadan, Iran; 314 Centre for Medical Informatics, University of Edinburgh, Edinburgh, UK; 315 Division of General Internal Medicine and Primary Care, Harvard University, Boston, Massachusetts, USA; 316 Center for Pediatric Trauma Research, Research Institute at Nationwide Children’s Hospital, Columbus, Ohio, USA; 317 National Institute of Infectious Diseases, Tokyo, Japan; 318 Finnish Institute of Occupational Health, Helsinki, Finland; 319 Institute of Medical Epidemiology, Martin Luther University Halle-Wittenberg, Halle, Germany; 320 Department of Medicine, University of Alabama at Birmingham, Birmingham, Alabama, USA; 321 Medicine Service, US Department of Veteran Affairs, Birmingham, Alabama, USA; 322 Department of Epidemiology, School of Preventive Oncology, Patna, India; 323 Department of Epidemiology, Healis Sekhsaria Institute for Public Health, Mumbai, India; 324 Department of Diseases and Noncommunicable Diseases and Health Promotion, Federal Ministry of Health, Brasilia, Brazil; 325 Hospital Universitario de la Princesa, Autonomous University of Madrid, Madrid, Spain; 326 Centro de Investigación Biomédica en Red Enfermedades Respiratorias (CIBERES), Madrid, Spain; 327 Department of Research Development, Federal Research Institute for Health Organization and Informatics of the Ministry of Health (FRIHOI), Moscow, Russia; 328 Hull York Medical School, University of Hull, Hull City, UK; 329 Usher Institute of Population Health Sciences and Informatics, University of Edinburgh, Edinburgh, UK; 330 Federal Research Institute for Health Organization and Informatics of the Ministry of Health (FRIHOI), Moscow, Russia; 331 Department of Psychiatry and Mental Health, University of Cape Town, Cape Town, South Africa; 332 Department of Psychology, Deakin University, Burwood, Victoria, Australia; 333 Department of Community Medicine, Ahmadu Bello University, Zaria, Nigeria; 334 Department of Anesthesiology & Pain Medicine, University of Washington, Seattle, Washington, USA; 335 Department of Criminology, Law and Society, University of California Irvine, Irvine, California, USA; 336 Department of Medicine, University of Valencia, Valencia, Spain; 337 Carlos III Health Institute, Biomedical Research Networking Center for Mental Health Network (CiberSAM), Madrid, Spain; 338 School of Social Work, University of Illinois, Urbana, Illinois, USA; 339 Department of Community Medicine, Iran University of Medical Sciences, Tehran, Iran; 340 Institute of Public Health, University of Gondar, Gondar, Ethiopia; 341 School of Public Health, University of Adelaide, Adelaide, South Australia, Australia; 342 School of Public Health, Post Graduate Institute of Medical Education and Research, Chandigarh, India; 343 Molecular Medicine and Pathology Department, University of Auckland, Auckland, New Zealand; 344 Clinical Hematology and Toxicology, Military Medical University, Hanoi, Vietnam; 345 Department of Health Economics, Hanoi Medical University, Hanoi, Vietnam; 346 Lee Kong Chian School of Medicine, Nanyang Technological University, Singapore; 347 Division of Health Sciences, University of Warwick, Coventry, UK; 348 Department of Community Medicine, University of Nigeria Nsukka, Enugu, Nigeria; 349 Argentine Society of Medicine, Buenos Aires, Argentina; 350 Velez Sarsfield Hospital, Buenos Aires, Argentina; 351 Central Research Institute of Cytology and Genetics, Federal Research Institute for Health Organization and Informatics of the Ministry of Health (FRIHOI), Moscow, Russia; 352 Department of Statistics, University of Brasília, Brasília, Brazil; 353 Directorate of Social Studies and Policies, Federal District Planning Company, Brasília, Brazil; 354 Raffles Neuroscience Centre, Raffles Hospital, Singapore; 355 Yong Loo Lin School of Medicine, National University of Singapore, Singapore; 356 Department of Medical and Surgical Sciences, University of Bologna, Bologna, Italy; 357 Occupational Health Unit, Sant’Orsola Malpighi Hospital, Bologna, Italy; 358 Department of Health Care Administration and Economics, National Research University Higher School of Economics, Moscow, Russia; 359 Foundation University Medical College, Foundation University, Islamabad, Pakistan; 360 Department of Psychiatry, University of São Paulo, São Paulo, Brazil; 361 Department of Psychology and Counselling, University of Melbourne, Melbourne, Victoria, Australia; 362 Department of Medicine, University of Melbourne, St Albans, Victoria, Australia; 363 Institute of Health and Society, University of Oslo, Oslo, Norway; 364 Department of Neurology, Technical University of Munich, Munich, Germany; 365 Centre for the Study of Regional Development, Jawahar Lal Nehru University, New Delhi, India; 366 Department of Preventive Medicine, Northwestern University, Chicago, Illinois, USA; 367 Centre for Suicide Research and Prevention, University of Hong Kong, Hong Kong, China; 368 Department of Social Work and Social Administration, University of Hong Kong, Hong Kong, China; 369 School of Allied Health Sciences, Addis Ababa University, Addis Ababa, Ethiopia; 370 Department of Psychopharmacology, National Center of Neurology and Psychiatry, Tokyo, Japan; 371 Health Economics & Finance, Jackson State University, Jackson, Mississippi, USA; 372 School of Medicine, Tsinghua University, Beijing, China; 373 Department of Epidemiology and Biostatistics, Wuhan University, Wuhan, China; 374 Global Health Institute, Wuhan University, Wuhan, China; 375 Department of Obstetrics & Gynaecology, A.C.S. Medical College and Hospital, Islamabad, Pakistan; 376 Department of Epidemiology, University Hospital of Setif, Setif, Algeria; 377 Maternal and Child Health Division, International Centre for Diarrhoeal Disease Research, Dhaka, Bangladesh; 378 Department of Medicine, Monash University, Melbourne, Victoria, Australia; 379 Student Research Committee, Babol University of Medical Sciences, Babol, Iran; 380 School of Public Health and Management, Chongqing Medical University, Chongqing, China; 381 Indian Institute of Public Health, Public Health Foundation of India, Gurugram, India; 382 Department of Health Metrics Sciences, School of Medicine, University of Washington, Seattle, Washington, USA; 383 University of Melbourne, Melbourne, Queensland, Australia

**Keywords:** epidemiology, descriptive epidemiology, burden of disease

## Abstract

**Background:**

The epidemiological transition of non-communicable diseases replacing infectious diseases as the main contributors to disease burden has been well documented in global health literature. Less focus, however, has been given to the relationship between sociodemographic changes and injury. The aim of this study was to examine the association between disability-adjusted life years (DALYs) from injury for 195 countries and territories at different levels along the development spectrum between 1990 and 2017 based on the Global Burden of Disease (GBD) 2017 estimates.

**Methods:**

Injury mortality was estimated using the GBD mortality database, corrections for garbage coding and CODEm—the cause of death ensemble modelling tool. Morbidity estimation was based on surveys and inpatient and outpatient data sets for 30 cause-of-injury with 47 nature-of-injury categories each. The Socio-demographic Index (SDI) is a composite indicator that includes lagged income per capita, average educational attainment over age 15 years and total fertility rate.

**Results:**

For many causes of injury, age-standardised DALY rates declined with increasing SDI, although road injury, interpersonal violence and self-harm did not follow this pattern. Particularly for self-harm opposing patterns were observed in regions with similar SDI levels. For road injuries, this effect was less pronounced.

**Conclusions:**

The overall global pattern is that of declining injury burden with increasing SDI. However, not all injuries follow this pattern, which suggests multiple underlying mechanisms influencing injury DALYs. There is a need for a detailed understanding of these patterns to help to inform national and global efforts to address injury-related health outcomes across the development spectrum.

## Introduction

Injury is an important cause of morbidity and mortality in nations at any point of the development spectrum. Previous research has shown that in 2015, injuries accounted for 11% of the global burden of disease, expressed in disability-adjusted life years (DALYs), with an estimated 973 million people sustaining injuries warranting some type of healthcare and 4.7 million deaths.^1^ Globally, since 1990, focused injury burden research has documented a declining trend in the burden of injury of all the major causes of injury.^2^

The epidemiological transition of non-communicable diseases (NCDs) replacing infectious diseases as the main contributors to disease burden has been well-documented.^1 3 4^ However, less focus has been given to the relationship between sociodemographic changes and injury outcomes. Up till now, few studies have been performed that studied the relationship between sociodemographic changes and overall injury rates. There have been reports on the associations of gross domestic product and unemployment with suicides, homicides, road injury and unintentional injuries.^5–12^ However, these studies focused on one specific cause of injury and on one type of injury outcome, mostly mortality. The findings of these studies indicated that the relationship between economic development and injury burden is not straightforward and mediated by many factors. A better understanding of this relationship may be achieved by investigating all causes of injury as well as looking at both fatal and non-fatal injury outcome.

Insight into the epidemiological transitions with regard to injuries can be achieved by a systematic analysis of the relationship between development and trends in mortality, incidence and burden of disease using a standardised approach. A systematic analysis may also reveal where health gains outpace or fall behind changes in development and allow for the identification of determinants and mediating factors of injury burden. This information allows identification determinants of injury burden. This information serves as a crucial input for guiding health system investments and priority-setting at the global, regional, national and subnational levels.

The Global Burden of Disease (GBD) 2015 study introduced a measure of development, the Socio-demographic Index (SDI). SDI combines information on income per capita, education and fertility. Comparisons between DALYs and SDI showed that age-standardised DALY rates for many communicable diseases declined profoundly over time, whereas improvements in SDI correlated strongly with the increasing importance of NCDs.^4^

This paper aims to provide an overview of injury mortality, incidence and DALYs from the GBD 2017 study, with detailed information on a range of causes of injuries; to examine the association between years of life lost (YLLs), YLDs and DALYs from injury and development, as measured by SDI, cause of injury, GBD region and over time; and to assess in which regions injury DALYs outpace or fall behind changes in development.

## Methods

### GBD 2017 study

The GBD 2017 study methods and results have been described in extensive detail elsewhere, including description of the analytical estimation framework used to measure deaths, YLLs, YLDs and DALYs.^4 13 14^ A summary overview of the GBD study is provided in online supplementary appendix 1. The methodological components specific to injuries estimation and SDI calculation are summarised below.

10.1136/injuryprev-2019-043296.supp1 Supplementary data

Injury incidence and death are defined as ICD-9 codes E800–E999 and ICD-10 chapters V–Y, except for deaths and cases of drug overdoses and unintentional alcohol poisoning, which are classified under drug and alcohol use disorders. These external cause-of-injury codes or ‘E codes’ are designated as mutually exclusive and collectively exhaustive within the injuries estimation process. In terms of the nature-of-injury codes (eg, the lower extremity amputation that can occur with a road injury), injuries were categorised into 47 mutually exclusive and collectively exhaustive nature-of-injury categories using chapters S and T in International Classification of Disease (ICD) ICD-10 and codes 800–999 in ICD-9 to quantify the various disabling outcomes of each cause of injury. Some injuries are trivial and unlikely to account for an important number of DALYs; hence, we only included injuries in our morbidity analysis that warranted some form of healthcare.

### Injury mortality and YLLs

The overall approach to estimate causes of death is provided in related publications.^13 15 16^ A summary is as follows. We first mapped data sources using different versions of ICD or alternative classification systems to the GBD cause list. These data sources included vital registration, verbal autopsy, mortality surveillance, censuses, surveys, hospitals, police records and mortuary data. We then made adjustments for ill-defined causes of death such that they mapped to an underlying cause of death. Next, we conducted ensemble models using GBD cause of death ensemble modelling (CODEm) software to estimate cause-specific mortality by age, sex, country, year and cause. CODEm is described in more detail elsewhere but in summary explores a large variety of possible models to estimate trends in causes of death using an algorithm to select varying combinations of covariates that are run through several modelling classes. The method then creates an ensemble of best-performing models that are determined by evaluating out-of-sample predictive validity. Deaths are then rescaled for each cause so that the sum equals the number of deaths from all causes to ensure internal consistency. YLLs were calculated by multiplying deaths by the residual life expectancy at the age of death based on the GBD 2017 standard model life table.^12^

### Injury incidence, prevalence and years lived with disability

Our method for estimating the incidence, prevalence and years lived with disability in non-fatal injury outcomes is provided in other GBD publications.^2 14^ A summary is as follows. We used DisMod-MR V.2.1 (a meta-regression tool for epidemiological modelling) to model injury incidence using data from emergency department and hospital records and survey data to produce cause-of-injury incidence by location, year, age and sex. Across every injury cause model, we used national income per capita as a covariate on excess mortality, which forces a negative relationship between income and mortality to take into account higher case fatality in lower-resource settings. After modelling incidence of each cause of injury, we used a severity hierarchy to identify the nature-of-injury category that would lead to the most long-term burden when an individual experiences multiple injuries. This hierarchy is based on pooled data sets of follow-up studies in which we translated each individual’s health status measure at 1 year after injury into a disability weight. This process is described in more detail in the GBD literature.^12 14 17–22^ Then, we generated matrices of the proportions of each cause of injury that are expected to lead to each nature of injury as determined in dual-coded (eg, both cause-of-injury and nature-of-injury coded) hospital and emergency department data sets and data from the Chinese National Injury Surveillance System.^23^ These data sets were used because the data were available in microdata format and they included dual-coded data in the format required for this specific part of the analysis. The resulting cause–nature matrices varied by injury warranting hospital admission versus injury warranting other healthcare, high-income/low-income countries, male/female and age group. In the next stage, we estimated short-term disability by cause and nature‐of‐injury category based on average duration for treated cases for each nature-of-injury category and for inpatient and outpatient injuries from the Dutch Injury Surveillance System.^17 18^ For 19 of the 47 nature-of-injury categories (eg, foreign body in ear, poisoning and fracture in ear), we supplemented these estimates with expert-driven estimates of short-term duration for nature-of-injury categories when the data set had insufficient information. For untreated injuries, the average factor by which the duration of short-term injury outcomes is increased for a given nature-of-injury category when the injury goes untreated was estimated.

For longer-term injuries, we calculated the proportion of injuries that would result in disability lasting more than a year for each nature-of-injury category by admission status and age. This calculation was based on an assumption that disability from injury affects all cases in the short term with a proportion having persistent disability 1 year after the injury greater than the pre-injury health status. These probabilities of developing permanent health loss were based on a pooled data set of seven large follow-up studies from China, the Netherlands and the USA that used patient-reported outcome measures to assess health status.^17–22 24 25^ We used the GBD healthcare access and quality (HAQ) index to estimate the ratio of treated to untreated injuries for each country–year grouping.^26^ The HAQ index is scaled from 0 to 100 and is based on 32 causes of death, covering a range of health service areas, which should not occur if effective care is present. Finally, we used DisMod-MR V.2.1 to compute the long-term prevalence (ie, 1 year or more) for each cause–nature combination from incidence, which also incorporated increased mortality risk of certain nature of injuries, such as traumatic brain injury based on meta-analyses of studies providing standardised mortality ratios of these conditions. YLDs were calculated as prevalence of a health state multiplied by a disability weight. These estimates were then corrected for comorbidity with other non-fatal diseases using methods described elsewhere in the GBD study.^13^

### Socio-demographic Index

SDI is a composite indicator that includes income per capita, average educational attainment over age 15 years and total fertility rate under age 25 years. The SDI has a value that ranges from 0 to 1. 0 represents the lowest income per capita, lowest educational attainmentand highest fertility under age 25 years observed across all GBD geographies from 1980 to 2017. 1 represents the highest income per capita, highest educational attainment and lowest fertility under 25 years observed across all GBD geographies from 1980 to 2017. The average relationship between YLLs, YLDs and YLDs divided by DALYs was calculated with SDI using Gaussian process regression modelling. We used these estimates of expected DALY rates that were predicted based on the full range of SDI to determine whether observed health patterns deviated from trends associated with changes along the development spectrum.

### GATHER compliance

This study complies with the GATHER (Guidelines for Accurate and Transparent Health Estimates Reporting) recommendations (online supplementary appendix 2).

10.1136/injuryprev-2019-043296.supp2 Supplementary data

## Results

### Mortality, incidence and burden of injury, 2017

In 2017, worldwide 55.9 million (95% Uncertainty Interval (UI) 55.4 to 56.5 million) people died. Of these deaths, 4.5 million (95% UI 4.3 to 4.6 million), 8.0% (95% UI 7.7% to 8.2%), were due to injuries. Major causes of injury deaths were road injury (27.7%), self-harm (17.7%), falls (15.5%) and interpersonal violence (9.0%).

There were 521 million (95% UI 493 to 548 million) cases of non-fatal injuries in 2017, representing an increase of 167 million from the 354 million (95% UI 338 to 372 million) cases of non-fatal injuries in 1990. The global age-standardised injury death rate was 57.9 per 100 000 (95% UI 55.9 to 59.2), with highest death rates for road injury (15.8 deaths per 100 000 (95% UI 15.2 to 16.3)), self-harm (10.0 deaths per 100 000 (95% UI 9.4 to 10.3)) and falls (9.2 deaths per 100 000 (95% UI 8.5 to 9.8)) (see online supplementary appendix table 1). Injury death rates were over twice as high in men compared with women (80.9 per 100 000 (95% UI 77.7 to 83.0) and 35.5 per 100 000 (95% UI 33.9 to 36.5), respectively). The global age-standardised injury incidence rate was 6762.6 per 100 000 (95% UI 6412.0 to 7118.1)), with highest incidence rates for falls (2237.6 new cases per 100 000 (95% UI 1989.7 to 2532.3)) and mechanical forces (943.6 new cases per 100 000 (95% UI 808.7 to 1100.6)) (see online supplementary appendix table 1). Injury incidence rates were almost twice as high in men compared with women (7827.1 per 100 000 (95% UI 7435.3 to 8242.9) and 5654.5 per 100 000 (95% UI 5351.3 to 5962.1), respectively).

10.1136/injuryprev-2019-043296.supp3 Supplementary data

Injuries contributed 10.1% (9.7%–10.5%) to the global burden of disease in 2017 (3267.0 DALYs per 100 000 (95% UI 3058.2 to 3505.1)). YLLs were responsible for the majority of the injury DALYs (77%; 2548 YLLs per 100 000 (95% UI 2462 to 2610)). The main contributors to injury DALYs were road injuries (871.1 DALYs per 100 000 (95% UI 827.9 to 917.3); 26.7%), falls (459.5 DALYs per 100 000 (95% UI 387.1 to 547.5); 14.1%), self-harm (429.0 per 100 000 (95% UI 401.6 to 443.5); 13.1%), interpersonal violence (334.3 DALYs per 100 000 (95% UI 304.7 to 360.5); 10.2%) and drowning (230.0 DALYs per 100 000 (95% UI 219.1 to 241.2); 7.0%) (see online supplementary appendix table 2). The injury burden was highest in Syria (16 341.1 DALYs per 100 000 (95% UI 15 892.7 to 16 858.4), Central African Republic (11 012.7 DALYs per 100 000 (95% UI 8807.9 to 12 913.8)) and Lesotho (7951.3 DALYs per 100 000 (95% UI 6424.8 to 9407.4)) and lowest in Maldives (1282.4 DALYs per 100 000 (95% UI 1138.1 to 1572.9)), Bermuda (1432.2 DALYs per 100 000 (95% UI 1267.5 to 1606.7)) and Italy (1458.1 DALYs per 100 000 (95% UI 1237.2 to 1739.4)) (see online supplementary appendix table 3. SDI level for each country in 2017 is also provided).

10.1136/injuryprev-2019-043296.supp4 Supplementary data

10.1136/injuryprev-2019-043296.supp5 Supplementary data

### Change over time

Between 1990 and 2017, the age-standardised injury DALY rates have declined from 4946 (95% UI 4655 to 5233) to 3267 DALYs (95% UI 3058 to 3505) per 100 000, with largest absolute declines in drowning (from 635 (95% UI 571 to 689) to 230 (95% UI 219 to 241) DALYs per 100 000), road injuries (from 1259 (95% UI 1182 to 1330) to 871 (95% UI 828 to 917) DALYs per 100 000), self-harm (from 687 (95% UI 621 to 723) to 429 (95% UI 402 to 443) DALYs per 100 000), and fire, heat and hot substances (from 197 (95% UI 157 to 228) to 111 (95% UI 93 to 129) DALYs per 100 000). Between 1990 and 2017, the age-standardised rates of YLDs and YLLs from injuries declined by 7.8% and 38.8%, respectively, while incidence of injuries only declined by 0.9%.

### Burden of injury by SDI level

The contribution of cause-of-injury category DALY rates to the total injury DALY rates differed by year, age category, sex and SDI level. The largest disparity in DALY rate by SDI level was found in 0–6 days olds, ranging from a high of 52 374 DALYs per 100 000 in the lowest SDI quintile to a low of 6109 DALYs per 100 000 in the highest SDI quintile. In men aged 15–49 years, conflict and terrorism stands out because of the high difference between highest and lowest DALY rates by level of SDI (countries with low SDI 496 DALYs (95% UI 414 to 589) per 100 000; countries with high SDI 2 DALYs (95% UI 1 to 2) per 100 000).

#### YLL and YLD rates by SDI level

**Figure 1:**
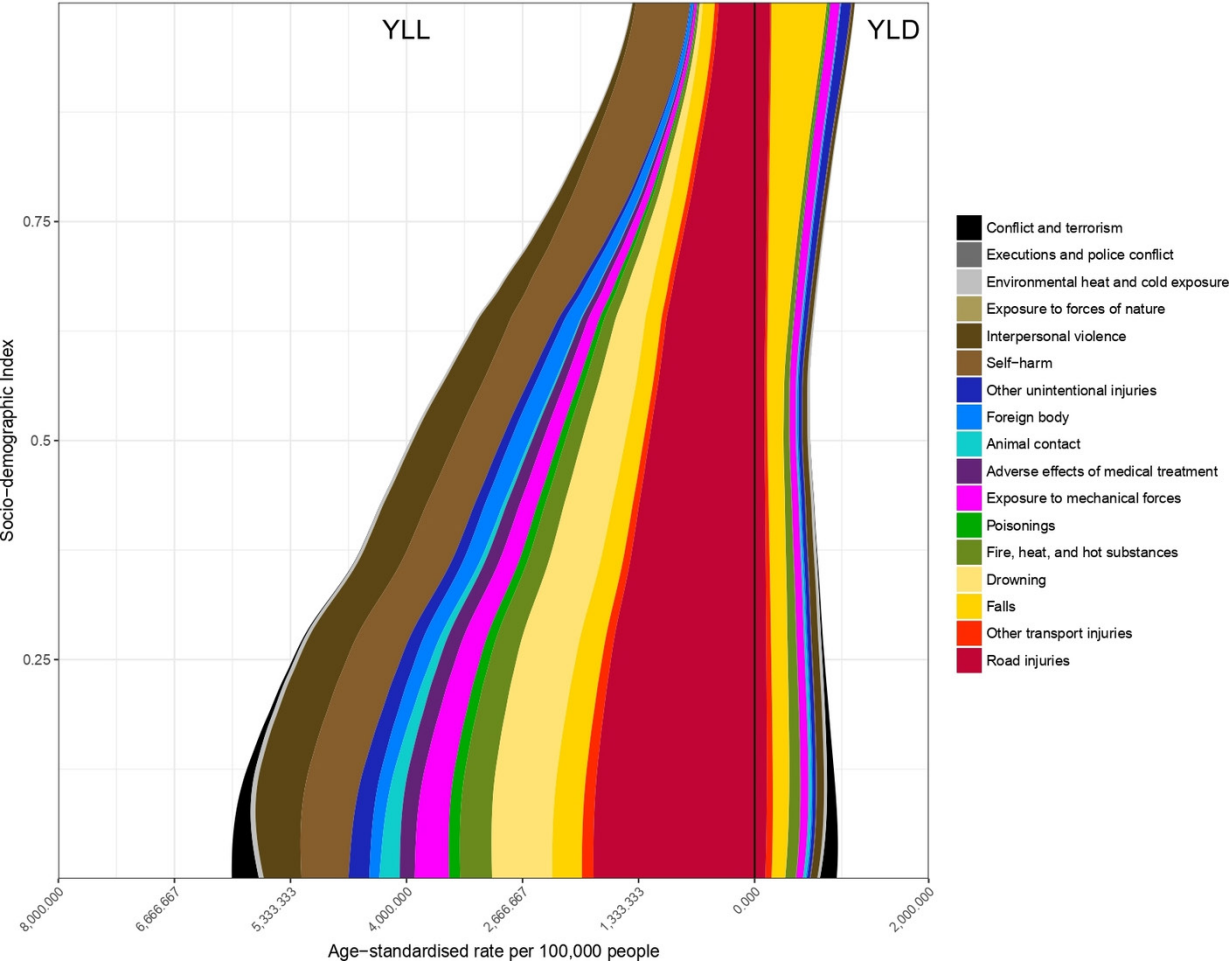
Age-standardised YLL and YLD rates for 17 cause-of-injury categories by level of Socio-demographic Index. YLL, years of life lost.

For many causes of injury, age-standardised YLL and YLD rates declined strikingly with increasing SDI, with proportionally largest decreases in YLL rates for conflict and terrorism (low SDI level 163.4 YLLs per 100 000; high SDI level 0.06 YLLs per 100 000), animal contact (low SDI level 140.0 YLLs per 100 000; high SDI level 2 YLLs per 100 000) and other unintentional injuries (low SDI level 7993 YLLs per 100 000; high SDI level 8.4 YLLs per 100 000). Figure 1 shows the level of age-standardised YLLs and YLDs per 100 000 against SDI (all regions, all years 1990–2017) by cause-of-injury. Largest decreases in YLD rates were seen for cause-of-injury categories conflict and terrorism, exposure to forces of nature and adverse effects of medical treatment. Exceptions were road injuries, self-harm and interpersonal violence. The age-standardised YLL rate of road injuries was highest at the low-middle range SDI levels and lowest at higher SDI levels, whereas YLDs from road injuries increased at higher SDI. The age-standardised road injuries YLL rate increased from low SDI to low-middle SDI, but declined at higher levels of SDI. For falls, at higher levels of SDI, the composition of the disease burden shifted towards YLDs as the primary driver of DALYs. YLLs made up 63%, 61% and 20% of DALYs from falls in low, middle and high SDI quintiles, respectively. For road injuries, the proportion of YLLs dropped from 91% in countries with low SDI to 70% in countries with high SDI.

#### Expected based on SDI versus observed burden of injury by SDI level, 1990–2017

**Figure 2:**
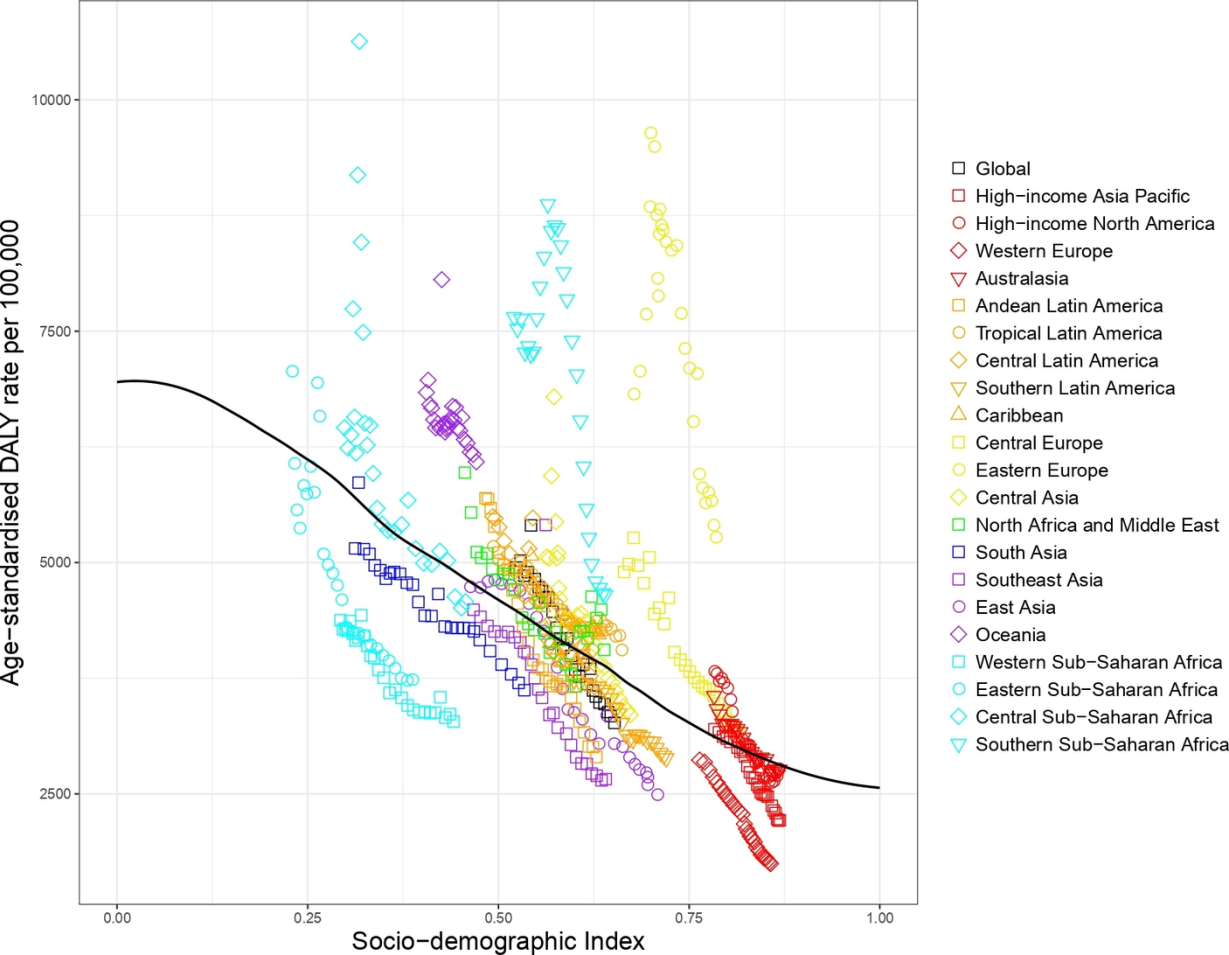
Co-evolution of all injury age-standardised DALY rates with SDI for the world and 21 GBD regions for 1990–2017 with comparison with the values expected on the basis of SDI alone. DALY, disability-adjusted life year; GBD, Global Burden of Disease; SDI, Socio-demographic Index.

Figure 2 shows the level of all injury age-standardised DALYs per 100 000 against SDI by GBD region from 1990 to 2017 in comparison with expected values (black line) based on SDI alone. The icons appearing above the black line for DALYs represent worse than expected injury DALYs and the icons appearing below represent better than expected injury DALYs. As SDI generally increases over time, successive markers represent years between 1990 and 2017. Regions where injury DALY rates were notably greater than expected based on SDI included Central and Southern Sub-Saharan Africa, Oceania, Eastern Europe, Central Europe and high-income North America. Regions where injury DALY rates were notably lower than expected based on SDI included Eastern and Western Sub-Saharan Africa, South Asia, Southeast Asia and Western Europe.

#### Road injury

**Figure 3:**
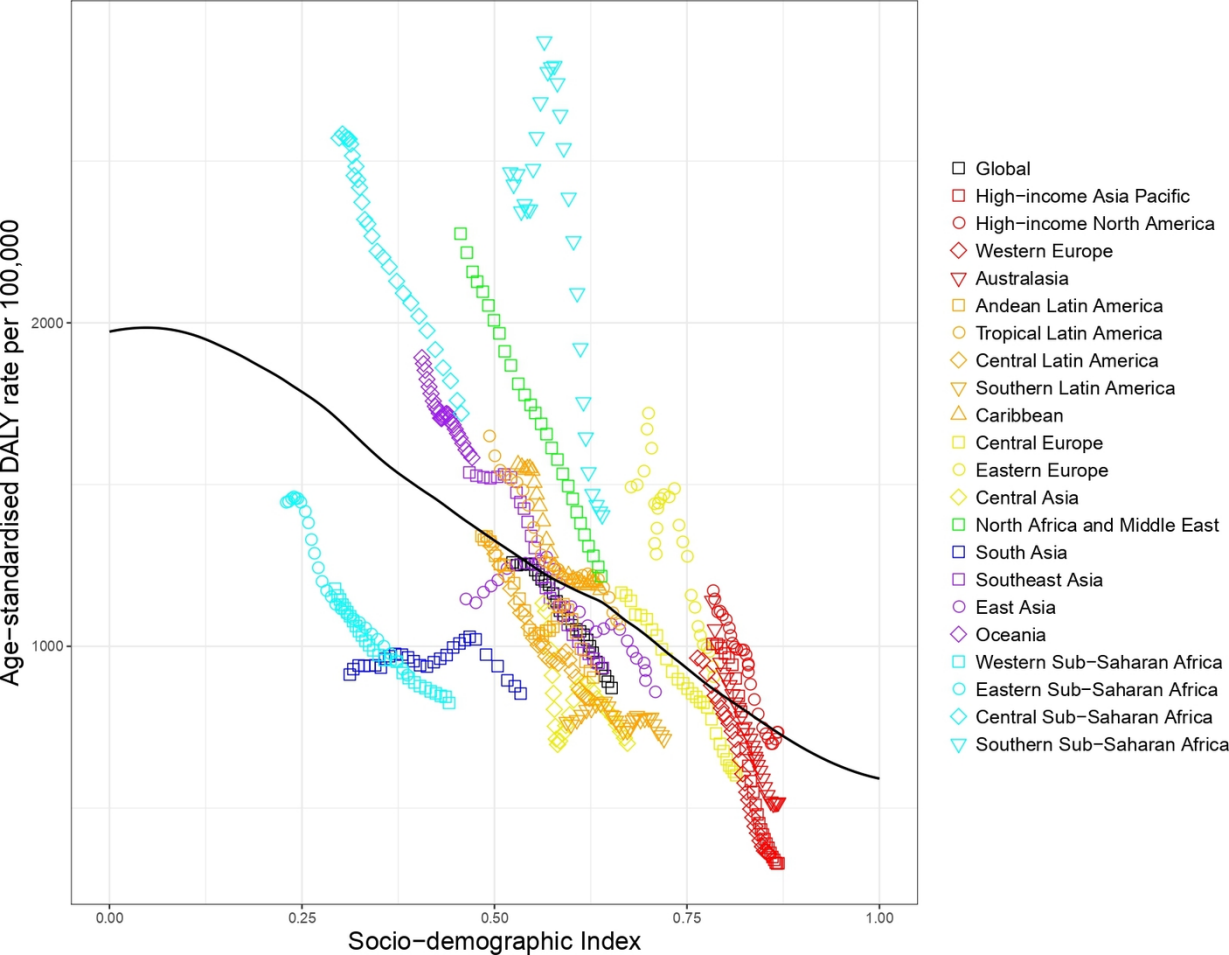
Co-evolution of road injury age-standardised DALY rates with SDI for the world and 21 GBD regions for 1990–2017 with comparison with the values expected on the basis of SDI alone. DALY, disability-adjusted life year; GBD, Global Burden of Disease; SDI, Socio-demographic Index.

The expected road injury DALY rate by SDI shows that most regions decreased in terms of road injury DALYs as SDI increased over time (see figure 3). South Asia, East Asia, Southern Sub-Saharan Africa and Eastern Europe are exceptions to this pattern, showing an initial increase and then a decline. In GBD 2017, the regions with worse than expected road injury DALYs based on SDI included North Africa and Middle East, Southern and Central Sub-Saharan Africa, Eastern Europe and Oceania, while regions with markedly better than expected rates included Eastern Sub-Saharan Africa, South Asia and Southern Latin America.

#### Interpersonal violence

**Figure 4:**
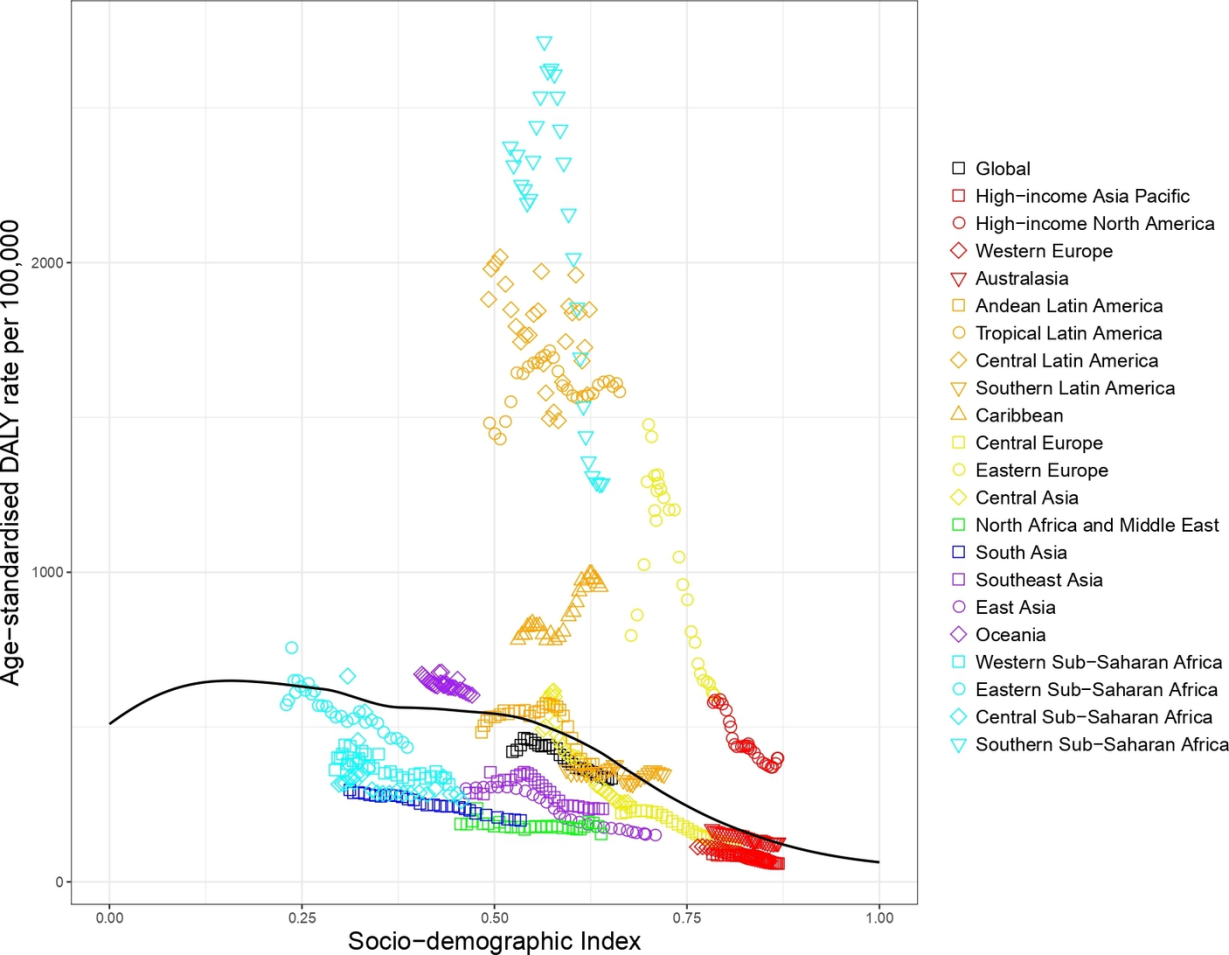
Co-evolution of interpersonal violence age-standardised DALY rates with SDI for the world and 21 GBD regions for 1990–2017 with comparison with the values expected on the basis of SDI alone. DALY, disability-adjusted life year; GBD, Global Burden of Disease; SDI, Socio-demographic Index.

In 2017, in all regions except for Southern Sub-Saharan Africa, Central Latin America, Tropical Latin America, Eastern Europe, Caribbean, Oceania and high-income North America, the observed interpersonal violence DALY rates were better than expected based on SDI (see figure 4). Between 1990 and 2017, in most regions with higher than expected DALYs, the gap between observed and expected interpersonal violence DALY rates decreased, except for Caribbean and Tropical Latin America, where the gap increased.

#### Self-harm

**Figure 5:**
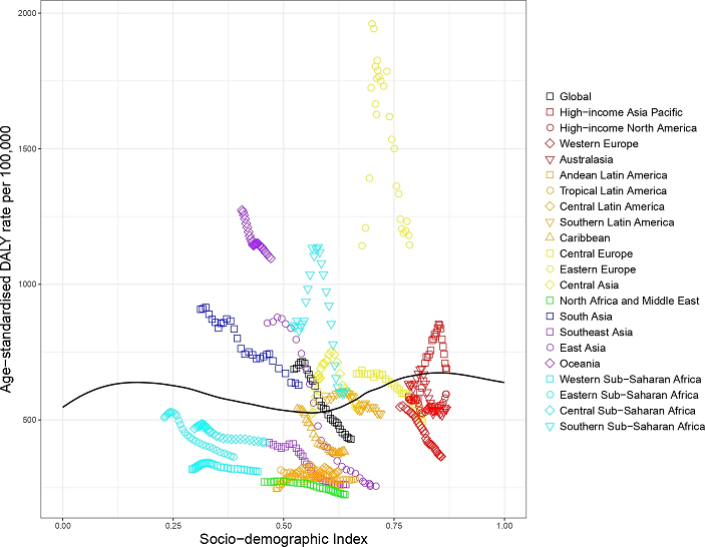
Co-evolution of self-harm age-standardised DALY rates with SDI for the world and 21 GBD regions for 1990–2017 with comparison with the values expected on the basis of SDI alone. DALY, disability-adjusted life year; GBD, Global Burden of Disease; SDI, Socio-demographic Index.

The patterns of observed and expected self-harm DALYs based on SDI by GBD regions between 1990 and 2017 differed markedly from those of other injuries (see figure 5). In 1990, observed self-harm DALY rates in East Asia and Eastern Europe were worse than expected based on SDI but rapidly declined over time, with observed DALY rates lower than expected in 2017. Southern Sub-Saharan Africa had worse than expected DALY rates but the other regions of Sub-Saharan Africa had better than expected DALY rates. North Africa and Middle East, Western Europe, Southeast Asia, and Andean, Central and Tropical Latin America all had better than expected DALY rates.

#### Drowning

**Figure 6:**
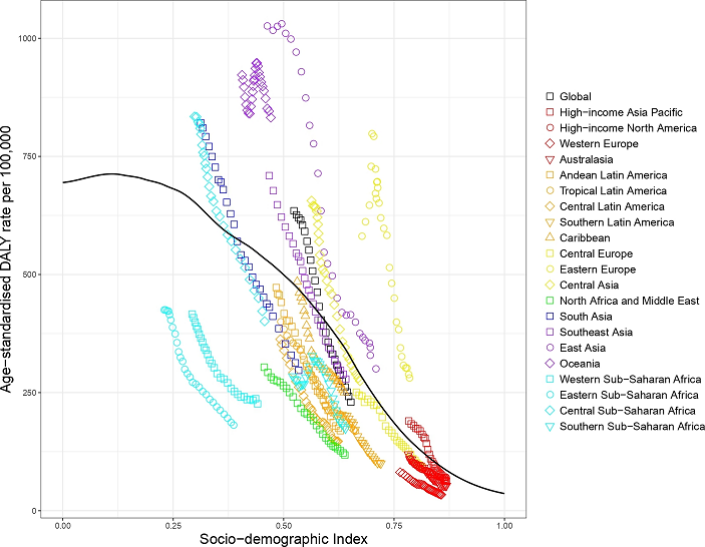
Co-evolution of drowning age-standardised DALY rates with SDI for the world and 21 GBD regions for 1990–2017 with comparison with the values expected on the basis of SDI alone. DALY, disability-adjusted life year; GBD, Global Burden of Disease; SDI, Socio-demographic Index.

Drowning DALY rates between 1990 and 2017 decreased in almost every GBD region regardless of their SDI value (figure 6), except for Oceania, Eastern Europe and Southern Sub-Saharan Africa. Eastern and Western Sub-Saharan Africa, North Africa and Middle East, Andean, Tropical, Central and Southern Latin America, Western Europe and Australasia had better than expected DALY rates, while Oceania, East Asia and Eastern Europe had worse than expected DALY rates based on SDI.

#### Falls

**Figure 7:**
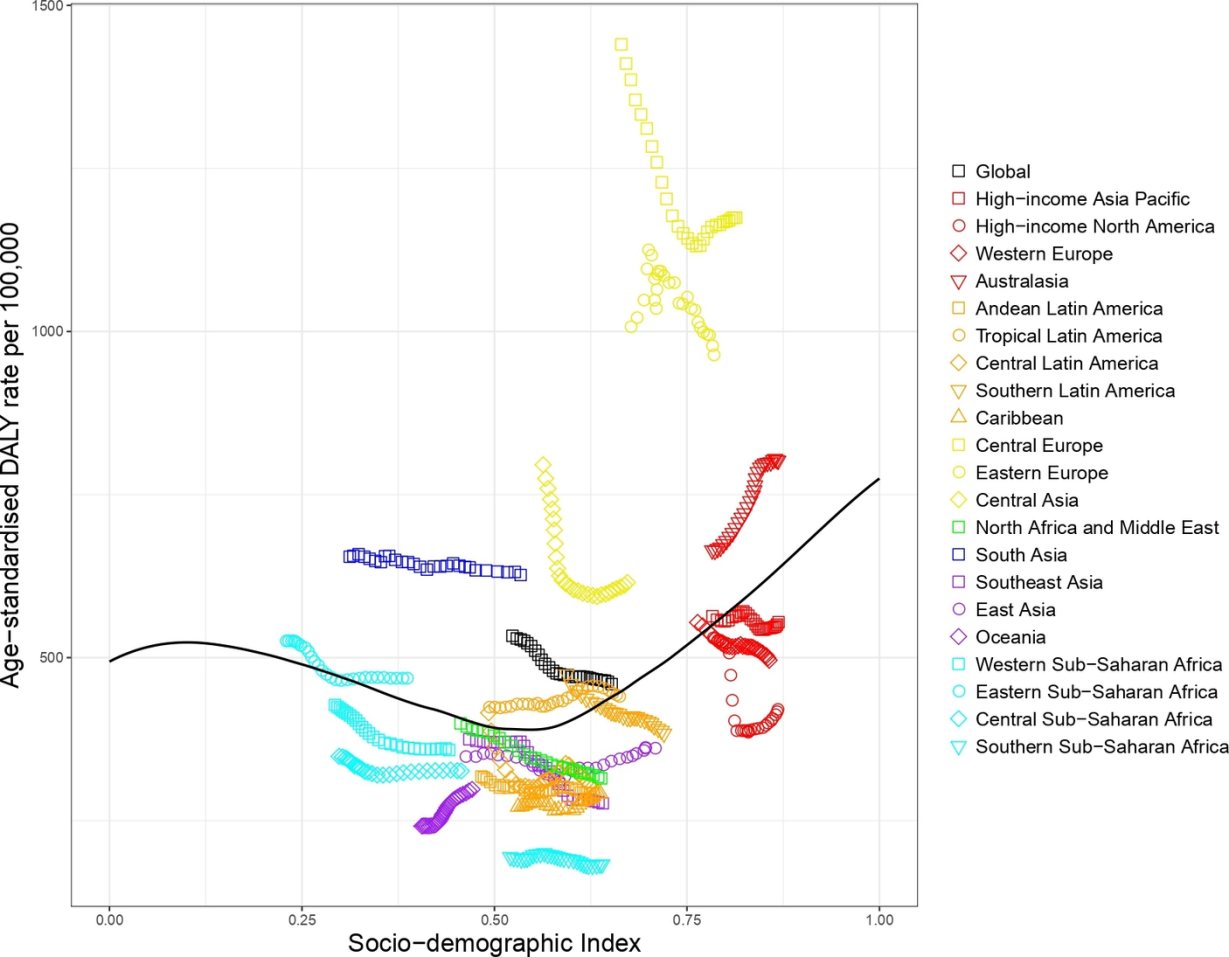
Co-evolution of falls age-standardised DALY rates with SDI for the world and 21 GBD regions for 1990–2017 with comparison with the values expected on the basis of SDI alone. DALY, disability-adjusted life year; GBD, Global Burden of Disease; SDI, Socio-demographic Index.

The patterns in falls globally followed more dynamic trends across regions as SDI increased from 1990 to 2017 (see figure 7). The regions that performed worse than expected in terms of SDI were Central Europe, Eastern Europe, South Asia, Central Asia and Australasia. Among these, Central Asia and Central Europe decreased and then increased, while Eastern Europe increased and then decreased. South Asia decreased steadily, while Australasia increased steadily until recent years. Among regions that performed better than expected, Oceania had increasing rates as SDI increased, while high-income North America dropped precipitously and then started increasing as SDI increased.

## Discussion

For many causes of injury, age-standardised DALY rates declined strikingly with increasing SDI, although road injury, interpersonal violence and self-harm did not strictly follow this pattern. Particularly for self-harm opposing patterns were observed in regions with similar SDI levels, for example, the trends in high-income Asia Pacific were opposite the trends in Western Europe, despite their proximity in terms of SDI. For road injuries, this effect was less pronounced; for nearly all regions, road injury DALY rates declined after 2005.

In Southern Sub-Saharan Africa, injury DALYs were worse than expected based on SDI in the overall injuries category as well as many of the specific injuries. In this region, road injury and interpersonal violence were important causes explaining the gap between observed and expected levels of overall injury DALYs. Many underlying and intertwining determinants of the high levels of interpersonal violence have been cited, including income inequality and poverty, high unemployment, rapid social change, corruption and poor rule of law, gender inequality, family breakdown, access to firearms, and alcohol and drug abuse.^27^ Despite these difficulties, however, and the worse-than-expected performance relative to SDI, our findings show that the DALY rates in Southern Sub-Saharan Africa have decreased from 2000 to 2017. This trend tallies with a reported declining number of injury deaths among young adults in South Africa.^28^

Of regions with a middle-high SDI, Eastern Europe stands out, because for most causes of injury, DALYs were much worse than expected based on SDI, particularly in the period 1990–2005. A compelling explanation for this finding may be the dissolution of the former Soviet Union and the resulting social and economic consequences on health and mortality.^29^ However, others have argued that causes of the health crisis are more complex and may result from a combination of historical and contemporary forces, including lifestyle habits, such as alcohol use, economic impoverishment, widening social inequality and the breakdown of political institutions.^30 31^ It should be noted that our study did aim to assess determinants of the burden of injury and caution is needed in attempting to draw conclusions with regard to possible reasons for regional trends and differences.

Another notable finding from our study was that for falls, at the higher levels of SDI, the composition of the disease burden shifted towards YLDs, rather than YLLs, as a more prominent driver of DALYs compared with areas with lower SDI. The proportion of DALYs due to YLDs also increases with higher levels of SDI among other injuries. It is possible that this shift in distribution reflects decreased mortality among injuries when people in higher SDI locations have access to better healthcare services. The shift in road injuries, for example, could be brought about by injury-prevention measures reducing the severity of the injury sustained (eg, seat belts and helmets) or by improved access to better quality care after an injury (eg, trauma systems). It is also possible that in age-standardised analyses, the shift towards YLDs may be due to the ageing of the population of countries with high SDI with commensurate age-related increases in injury incidence. For example, the incidence of falls increases substantially with age and most of the burden from falls in high-SDI countries occurs in the very old.^32^

### Limitations

Our analysis has several limitations. First, as SDI and time are correlated, we may be over interpreting SDI as a driver of change as it could well be driven largely by other factors changing over time, not necessarily linked to SDI, such as climate change.

Second, limited data are available to quantify burden of injuries in the world. Major limitations of the cause-of-death data are low or absent coverage of vital registration or verbal autopsy data in many parts of the world, incompleteness of death certification systems and differences in the proportion of injury deaths classified in ill-defined codes.^33–36^ Few data were available for non-fatal injuries, and if data were available, injury was frequently recorded as a mix of cause and nature-of-injury codes and often a preponderance of nature-of-injury codes, while our analyses require attributing health outcomes to cause of injury. As a result, many non-fatal injury hospitals and emergency departments data sets could not be used. Furthermore, short-term duration of several nature-of-injury categories was based on expert-driven estimates because patient data was not available. Besides, gathering data on deaths and morbidity due to forces of nature (ie, disasters) and collective violence is complicated by the fact that their aftereffects may severely disrupt the infrastructure of vital and health registration systems.^37^ The statistical methods that we have used to assess mortality, incidence and prevalence can borrow strength over time and geography to ensure an estimate for all causes and all countries. Nevertheless, estimates for populations and time periods with few or absent data are inherently less precise.

Non-fatal injuries are reported by both cause of injury and nature of injury. Since our model requires a one-to-one relationship between cause-of-injury and nature-of-injury category, we developed a nature-of-injuries severity hierarchy that selects the injury that was likely to be responsible for the largest burden in a person with more than one injury. This means that we ignore the other injuries sustained by such individuals and this may have led to some underestimation of the burden of non-fatal injury. We decided to use such a hierarchy after it proved difficult to use statistical methods on sparse data to parse estimates across co-occurring injuries.

A second methodological limitation is the assessment of the probability of permanent health loss, one of the main drivers of non-fatal burden of disease. The probability of long-term injury was based on patient-reported outcome data from follow-up studies in just three countries. Also, long-term patient-reported outcome data may be influenced by response shift bias. Response shift is a change of outcome due to a change of the measurement perspective of the respondent (‘internal measurement scale’), where the usual change is towards adaptation. In our study, response shift may have resulted in an underestimation of the severity of long-term consequences of injury and consequently, to an underestimation of the non-fatal burden of injury.

Third, even though a strong correlation between SDI and injury DALYs, YLLs and YLDs was found, this cannot be interpreted as being causal in nature, because income per capita and education, two of the three components of SDI, were also used as covariates in all of the injury models except exposure to forces of nature and collective violence and legal intervention. In its original formulation, Murray *et al* suggested that SDI utility may be improved in the future through consideration of additional societal elements, such as inequality in each component.^1^

## Conclusions

The overall pattern is that of declining injury burden with increasing development. Not all injuries follow this pattern, suggesting that there are multiple underlying mechanisms influencing injury outcomes. The detailed understanding of these patterns helps to inform countries how best to respond to changes in injury outcomes that occur with development and, in case of countries where health gains outpace development, may help to identify which prevention and/or healthcare measures have been taken in these countries.

**What is already known on the subject**

- Morbidity and mortality from injuries are known to be affected by socioeconomic development.

**What this study adds**

- This study provides more recent estimates of global morbidity and mortality from injuries with a greater level of detail than has previously been reported and with an updated method for measuring sociodemographic development.
- This study found that many injuries decreased in terms of morbidity and mortality as sociodemographic development increased over time, but also identified important exceptions to this trend.
- The study adds to the body of discussion on how economic development and sociodemographic changes should be considered in preventing future injury burden.
