# Correction: *Burden of injury along the development spectrum: associations between the Socio-demographic Index and disability-adjusted life year estimates from the Global Burden of Disease Study 2017*

**DOI:** 10.1136/injuryprev-2019-043296corr1

**Published:** 2020-09-28

Haagsma JA, James SL, Castle CD, *et al*. Burden of injury along the development spectrum: associations between the Socio-demographic Index and disability-adjusted life year estimates from the Global Burden of Disease Study 2017. *Inj Prev* 2020. doi: 10.1136/injuryprev-2019-043296

This article was previously published with errors in authorship.

Author Rakhi Dandona has been added prior to author Ahmad Daryani. Affiliations for Rakhi Dandona are below:

Rakhi Dandona^2, 382, 384^

2 Institute for Health Metrics and Evaluation, University of Washington, Seattle, Washington, USA

382 Department of Health Metrics Sciences, School of Medicine, University of Washington, Seattle, Washington, USA

384 Public Health Foundation of India, Gurugram, India.

