# Supplementary material for: Burden of injury along the development spectrum: associations between the Socio-demographic Index and disability-adjusted life year estimates from the Global Burden of Disease Study 2017
Source: Inj Prev. 2020 Jan 8;26(Suppl 1):i12–26. doi: 10.1136/injuryprev-2019-043296 (PMC7571356; doi:10.1136/injuryprev-2019-043296)
Supplement: Supplementary data [file injuryprev-2019-043296supp003.pdf]

Table 1: Age-standardized mortality and incidence rates in 2017 and percentage change from 1990 to 2017 by cause of injury				
Cause	Deaths (95% UI)		Incidence (95% UI)	
	2017 age-standardised rates per 100,000	Percentage change in age-standardised rates between 1990 and 2017	2017 age-standardised rates per 100,000	Percentage change in age-standardised rates between 1990 and 2017
**All injuries**	**57.9 (55.9 to 59.2)**	**-31.6% (-33.6% to -29.1%)**	**6 763 (6 412 to 7 118)**	**-0.9% (-2.3% to 0.6%)**
**Transport injuries**	**17.0 (16.4 to 17.4)**	**-28.6% (-33.2% to -24.8%)**	**823 (732 to 923)**	**8.3% (4.3% to 12.2%)**
Road injuries	15.8 (15.2 to 16.3)	-29.0% (-33.6% to -25.0%)	692 (605 to 786)	11.3% (6.4% to 15.8%)
Pedestrian road injuries	6.2 (5.9 to 6.8)	-37.1% (-44.2% to -29.9%)	141 (116 to 168)	3.0% (-3.4% to 9.5%)
Cyclist road injuries	0.9 (0.7 to 1.0)	7.7% (-12.6% to 31.4%)	152 (124 to 187)	24.1% (17.0% to 31.6%)
Motorcyclist road injuries	2.9 (2.5 to 3.0)	-11.1% (-29.5% to 1.3%)	129 (105 to 157)	30.0% (22.2% to 37.4%)
Motor vehicle road injuries	5.8 (5.4 to 6.0)	-30.0% (-36.2% to -23.8%)	207 (173 to 247)	-4.9% (-10.5% to 0.9%)
Other road injuries	0.1 (0.1 to 0.2)	-27.9% (-40.5% to 13.3%)	63 (49 to 80)	38.0% (30.5% to 45.4%)
Other transport injuries	1.2 (1.1 to 1.4)	-22.1% (-32.0% to -9.9%)	131 (109 to 158)	-5.1% (-8.8% to -1.1%)
**Unintentional injuries**	**23.8 (22.4 to 24.7)**	**-35.5% (-38.1% to -31.3%)**	**5 400 (5 075 to 5 739)**	**-1.4% (-3.2% to 0.3%)**
Falls	9.2 (8.5 to 9.8)	-5.9% (-13.7% to 3.5%)	2 238 (1 990 to 2 532)	-3.7% (-7.4% to -0.3%)
Drowning	4.0 (3.8 to 4.1)	-57.4% (-60.1% to -53.3%)	5 (4 to 5)	-27.7% (-32.0% to -22.5%)
Fire, heat, and hot substances	1.6 (1.3 to 1.7)	-46.6% (-49.7% to -38.8%)	119 (99 to 142)	-5.4% (-11.1% to 0.3%)
Poisonings	0.9 (0.7 to 1.0)	-37.4% (-56.9% to -20.3%)	55 (44 to 68)	6.0% (0.5% to 11.4%)
Poisoning by carbon monoxide	0.5 (0.3 to 0.5)	-41.2% (-60.8% to -28.9%)	14 (10 to 19)	6.3% (-2.4% to 14.8%)
Poisoning by other means	0.5 (0.4 to 0.5)	-33.5% (-53.2% to -10.5%)	41 (32 to 52)	5.9% (-0.2% to 11.9%)
Exposure to mechanical forces	1.8 (1.5 to 1.8)	-36.2% (-47.8% to -30.5%)	944 (809 to 1 101)	-1.7% (-5.5% to 2.0%)
Unintentional firearm injuries	0.3 (0.3 to 0.3)	-46.3% (-51.7% to -37.7%)	25 (18 to 34)	-3.6% (-10.2% to 2.9%)
Other exposure to mechanical forces	1.5 (1.2 to 1.6)	-33.7% (-48.3% to -27.0%)	918 (788 to 1 073)	-1.7% (-5.5% to 2.1%)
Adverse effects of medical treatment	1.6 (1.4 to 1.8)	-25.4% (-30.3% to -16.3%)	437 (376 to 502)	41.9% (34.8% to 49.3%)
Animal contact	1.1 (0.6 to 1.2)	-35.1% (-43.8% to -19.3%)	574 (492 to 671)	-7.1% (-9.7% to -4.3%)
Venomous animal contact	0.9 (0.5 to 1.1)	-34.0% (-44.5% to -16.6%)	275 (225 to 334)	-0.6% (-4.4% to 3.4%)
Non-venomous animal contact	0.1 (0.1 to 0.2)	-41.5% (-55.6% to -15.2%)	299 (244 to 363)	-12.3% (-14.8% to -9.8%)
Foreign body	1.7 (1.6 to 1.8)	-34.8% (-39.4% to -30.8%)	303 (262 to 348)	-0.4% (-2.2% to 1.5%)
Pulmonary aspiration and foreign body in airway	1.6 (1.5 to 1.7)	-32.3% (-36.7% to -28.3%)	18 (15 to 22)	9.7% (4.4% to 14.9%)
Foreign body in eyes	--	--	230 (191 to 273)	-1.8% (-3.9% to 0.4%)
Foreign body in other body part	0.1 (0.1 to 0.1)	-56.9% (-64.3% to -31.6%)	55 (45 to 67)	2.8% (0.3% to 5.4%)
Environmental heat and cold exposure	0.7 (0.5 to 0.8)	-47.3% (-52.9% to -44.1%)	115 (96 to 137)	-1.5% (-5.3% to 1.7%)
Exposure to forces of nature	0.1 (0.1 to 0.1)	-86.6% (-88.7% to -84.0%)	9 (8 to 11)	-83.1% (-85.0% to -80.7%)
Other unintentional injuries	1.2 (1.2 to 1.3)	-39.8% (-43.3% to -35.0%)	602 (512 to 699)	-1.2% (-5.2% to 2.6%)
**Self-harm and interpersonal violence**	**17.1 (16.3 to 17.5)**	**-28.8% (-32.2% to -24.9%)**	**540 (487 to 596)**	**-8.0% (-10.9% to -4.4%)**
Self-harm	10.0 (9.4 to 10.3)	-35.4% (-39.5% to -30.4%)	50 (42 to 59)	-16.3% (-21.4% to -11.5%)
Self-harm by firearm	0.8 (0.7 to 1.0)	-34.6% (-38.2% to -30.4%)	1 (0 to 1)	-29.3% (-37.0% to -21.6%)
Self-harm by other specified means	9.2 (8.5 to 9.5)	-35.4% (-39.8% to -30.2%)	50 (42 to 58)	-16.2% (-21.2% to -11.3%)
Interpersonal violence	5.2 (4.7 to 5.5)	-22.0% (-24.9% to -18.9%)	296 (253 to 345)	-6.5% (-10.6% to -2.4%)
Assault by firearm	2.2 (1.9 to 2.4)	-0.4% (-5.6% to 5.3%)	7 (5 to 9)	7.2% (-0.2% to 15.0%)
Assault by sharp object	1.2 (0.9 to 1.4)	-34.1% (-38.6% to -25.8%)	55 (43 to 70)	-8.5% (-14.3% to -2.7%)
Assault by other means	1.8 (1.6 to 2.1)	-32.3% (-39.2% to -25.2%)	234 (200 to 274)	-6.4% (-10.5% to -1.9%)
Conflict and terrorism	1.7 (1.6 to 1.9)	-3.4% (-12.0% to 6.6%)	167 (143 to 202)	-14.1% (-19.3% to -5.3%)

Cause	Deaths (95% UI)		Incidence (95% UI)	
	2017 age-standardised rates per 100,000	Percentage change in age-standardised rates between 1990 and 2017	2017 age-standardised rates per 100,000	Percentage change in age-standardised rates between 1990 and 2017
Executions and police conflict	0.2<br>(0.2 to 0.2)	64.6%<br>(48.2% to 208.1%)	26<br>(22 to 32)	72.6%<br>(50.4% to 247.9%)
