# Supplementary material for: Burden of injury along the development spectrum: associations between the Socio-demographic Index and disability-adjusted life year estimates from the Global Burden of Disease Study 2017
Source: Inj Prev. 2020 Jan 8;26(Suppl 1):i12–26. doi: 10.1136/injuryprev-2019-043296 (PMC7571356; doi:10.1136/injuryprev-2019-043296)
Supplement: Supplementary data [file injuryprev-2019-043296supp004.pdf]

Table 2: Age-standardized YLL, YLD, and DALY rates in 2017 and percentage change from 1990 to 2017 by cause of injury

Cause	YLLs (95% UI)		YLDs (95% UI)		DALYs (95% UI)	
	2017 age-standardised rates per 100,000	Percentage change in age-standardised rates between 1990 and 2017	2017 age-standardised rates per 100,000	Percentage change in age-standardised rates between 1990 and 2017	2017 age-standardised rates per 100,000	Percentage change in age-standardised rates between 1990 and 2017
**All injuries**	**2 548 (2 462 to 2 610)**	**-38.8% (-40.9% to -35.9%)**	**719 (529 to 948)**	**-7.8% (-9.3% to -6.3%)**	**3 267 (3 058 to 3 505)**	**-34.0% (-36.4% to -31.0%)**
**Transport injuries**	**800 (776 to 823)**	**-34.0% (-37.9% to -30.1%)**	**167 (120 to 223)**	**-2.9% (-4.8% to -0.9%)**	**968 (914 to 1 024)**	**-30.1% (-34.1% to -26.4%)**
Road injuries	745 (718 to 767)	-34.4% (-38.5% to -30.4%)	126 (90 to 169)	2.2% (0.3% to 4.0%)	871 (828 to 917)	-30.8% (-35.0% to -26.9%)
Pedestrian road injuries	270 (254 to 301)	-44.8% (-51.3% to -37.2%)	34 (24 to 46)	-5.4% (-8.0% to -2.9%)	304 (284 to 332)	-42.2% (-48.6% to -35.0%)
Cyclist road injuries	36 (32 to 41)	-1.8% (-23.2% to 22.7%)	21 (15 to 29)	12.0% (8.7% to 15.1%)	57 (49 to 66)	2.8% (-12.4% to 18.7%)
Motorcyclist road injuries	146 (128 to 155)	-16.1% (-32.1% to -4.4%)	32 (22 to 43)	17.9% (15.1% to 20.8%)	178 (157 to 192)	-11.5% (-26.9% to -1.1%)
Motor vehicle road injuries	285 (268 to 300)	-32.8% (-37.7% to -24.7%)	32 (23 to 43)	-11.9% (-13.4% to -10.4%)	317 (298 to 335)	-31.1% (-35.8% to -23.6%)
Other road injuries	7 (6 to 8)	-32.7% (-45.6% to 10.7%)	8 (5 to 11)	31.2% (27.7% to 34.2%)	14 (12 to 17)	-9.0% (-23.0% to 19.3%)
Other transport injuries	55 (51 to 65)	-27.6% (-36.9% to -14.8%)	41 (29 to 55)	-15.7% (-17.7% to -13.4%)	96 (83 to 112)	-23.0% (-29.4% to -14.9%)
**Unintentional injuries**	**929 (866 to 969)**	**-48.4% (-51.4% to -43.3%)**	**460 (333 to 618)**	**-9.9% (-11.5% to -8.3%)**	**1 389 (1 241 to 1 560)**	**-39.9% (-43.5% to -35.1%)**
Falls	217 (196 to 229)	-18.5% (-31.7% to -6.2%)	243 (173 to 330)	-9.3% (-10.7% to -7.9%)	459 (387 to 547)	-13.9% (-21.3% to -8.0%)
Drowning	228 (217 to 240)	-63.9% (-66.6% to -59.8%)	2 (1 to 2)	-35.7% (-39.0% to -32.3%)	230 (219 to 241)	-63.8% (-66.5% to -59.7%)
Fire, heat, and hot substances	71 (58 to 79)	-50.8% (-55.8% to -39.3%)	40 (28 to 55)	-24.4% (-29.4% to -19.3%)	111 (93 to 129)	-43.7% (-49.3% to -34.1%)
Poisonings	44 (33 to 49)	-44.4% (-61.5% to -26.3%)	6 (4 to 8)	-8.1% (-9.8% to -6.1%)	50 (39 to 56)	-41.6% (-57.9% to -24.5%)
Poisoning by carbon monoxide	19 (14 to 21)	-48.0% (-65.2% to -36.0%)	1 (1 to 1)	-0.3% (-3.4% to 2.9%)	20 (15 to 22)	-46.8% (-63.8% to -35.1%)
Poisoning by other means	25 (19 to 28)	-41.3% (-58.5% to -16.5%)	5 (3 to 7)	-9.4% (-11.6% to -7.2%)	30 (24 to 34)	-37.7% (-53.7% to -15.3%)
Exposure to mechanical forces	84 (72 to 88)	-40.9% (-51.5% to -35.5%)	62 (43 to 87)	-9.8% (-11.5% to -8.1%)	146 (124 to 173)	-30.8% (-38.4% to -25.8%)
Unintentional firearm injuries	14 (13 to 17)	-48.5% (-53.7% to -39.0%)	4 (3 to 5)	-9.3% (-10.7% to -8.0%)	18 (17 to 21)	-43.1% (-48.2% to -34.5%)
Other exposure to mechanical forces	70 (58 to 74)	-39.1% (-52.5% to -32.4%)	58 (40 to 82)	-9.8% (-11.6% to -8.1%)	128 (107 to 152)	-28.5% (-38.0% to -23.1%)
Adverse effects of medical treatment	58 (48 to 71)	-28.7% (-37.2% to -14.6%)	4 (3 to 7)	41.8% (34.7% to 49.2%)	63 (52 to 75)	-26.1% (-35.1% to -11.7%)
Animal contact	52 (29 to 62)	-39.0% (-50.2% to -20.5%)	14 (9 to 19)	-16.1% (-17.8% to -14.7%)	66 (42 to 77)	-35.3% (-45.5% to -19.8%)
Venomous animal contact	46 (23 to 55)	-38.0% (-51.0% to -18.1%)	9 (6 to 13)	-11.3% (-13.0% to -9.5%)	55 (33 to 65)	-34.7% (-46.0% to -17.4%)
Non-venomous animal contact	7 (5 to 10)	-44.7% (-61.9% to -13.7%)	5 (3 to 7)	-24.4% (-26.8% to -22.4%)	11 (8 to 15)	-38.1% (-54.7% to -18.5%)
Foreign body	83 (78 to 89)	-46.7% (-51.4% to -41.9%)	12 (9 to 16)	-10.0% (-12.6% to -7.2%)	95 (88 to 102)	-43.8% (-48.3% to -39.0%)
Pulmonary aspiration and foreign body in airway	78 (73 to 84)	-44.2% (-48.7% to -39.0%)	2 (1 to 3)	-9.1% (-14.6% to -3.1%)	80 (75 to 85)	-43.6% (-48.2% to -38.5%)
Foreign body in eyes	--	--	3 (1 to 4)	-7.0% (-10.6% to -4.5%)	3 (1 to 4)	-7.0% (-10.6% to -4.5%)
Foreign body in other body part	5 (4 to 7)	-68.6% (-75.3% to -46.8%)	8 (5 to 10)	-11.2% (-13.7% to -8.5%)	13 (10 to 15)	-49.0% (-57.2% to -29.6%)
Environmental heat and cold exposure	24 (16 to 27)	-51.8% (-55.4% to -47.9%)	19 (14 to 26)	-12.5% (-15.3% to -9.7%)	43 (32 to 51)	-39.7% (-43.7% to -35.7%)
Exposure to forces of nature	6 (6 to 7)	-88.1% (-90.1% to -85.6%)	9 (7 to 12)	176.3% (155.6% to 195.3%)	16 (13 to 19)	-72.6% (-78.1% to -65.5%)
Other unintentional injuries	61 (59 to 64)	-44.4% (-48.4% to -39.0%)	49 (34 to 69)	-9.0% (-10.6% to -7.5%)	110 (95 to 130)	-32.8% (-37.4% to -27.9%)
**Self-harm and interpersonal violence**	**819 (782 to 843)**	**-29.1% (-32.3% to -25.5%)**	**91 (71 to 114)**	**-5.5% (-8.1% to -2.6%)**	**910 (872 to 944)**	**-27.3% (-30.3% to -23.7%)**
Self-harm	424 (397 to 438)	-37.7% (-41.8% to -32.9%)	5 (4 to 7)	-26.2% (-28.6% to -23.7%)	429 (402 to 443)	-37.6% (-41.7% to -32.8%)
Self-harm by firearm	33 (28 to 42)	-35.0% (-38.6% to -29.8%)	0 (0 to 0)	-31.5% (-33.8% to -28.9%)	34 (28 to 42)	-35.0% (-38.6% to -29.8%)
Self-harm by other specified means	390 (364 to 405)	-37.9% (-42.3% to -32.8%)	5 (4 to 7)	-26.1% (-28.6% to -23.6%)	395 (369 to 410)	-37.8% (-42.1% to -32.8%)
Interpersonal violence	277 (248 to 294)	-22.6% (-25.7% to -19.0%)	57 (44 to 73)	-8.1% (-9.8% to -6.3%)	334 (305 to 361)	-20.5% (-23.3% to -17.2%)
Assault by firearm	123 (104 to 132)	1.0% (-5.0% to 6.5%)	1 (1 to 2)	1.7% (0.2% to 3.0%)	124 (106 to 134)	1.0% (-5.0% to 6.4%)
Assault by sharp object	59 (48 to 72)	-35.4% (-40.0% to -26.6%)	6 (4 to 8)	-15.0% (-17.1% to -12.7%)	65 (53 to 79)	-34.0% (-38.5% to -25.8%)
Sexual violence	--	--	27 (18 to 40)	-1.4% (-3.1% to 0.3%)	27 (18 to 40)	-1.4% (-3.1% to 0.3%)
Assault by other means	95 (83 to 112)	-34.4% (-40.2% to -27.5%)	23 (16 to 30)	-14.0% (-16.1% to -12.0%)	117 (106 to 134)	-31.2% (-36.4% to -24.9%)
Conflict and terrorism	107 (98 to 119)	-3.2% (-12.1% to 7.6%)	27 (18 to 40)	4.8% (-3.4% to 12.7%)	134 (119 to 152)	-1.7% (-9.0% to 7.0%)
Executions and police conflict	11 (11 to 12)	69.2% (51.4% to 222.2%)	2 (1 to 2)	40.1% (26.1% to 69.0%)	13 (13 to 14)	64.7% (48.8% to 191.5%)
