# Supplementary material for: Burden of injury along the development spectrum: associations between the Socio-demographic Index and disability-adjusted life year estimates from the Global Burden of Disease Study 2017
Source: Inj Prev. 2020 Jan 8;26(Suppl 1):i12–26. doi: 10.1136/injuryprev-2019-043296 (PMC7571356; doi:10.1136/injuryprev-2019-043296)
Supplement: Supplementary data [file injuryprev-2019-043296supp005.pdf]

Table 3: Age-standardised mortality, YLL, YLD, and DALY rates in 2017 and percentage change from 1990 to 2017 for all injuries by country

SDI Quintile	Location	Deaths (95% UI)		YLLs (95% UI)		YLDs (95% UI)		DALYs (95% UI)	
		2017 age-standardised rates per 100,000	Percentage change in age-standardised rates between 1990 and 2017	2017 age-standardised rates per 100,000	Percentage change in age-standardised rates between 1990 and 2017	2017 age-standardised rates per 100,000	Percentage change in age-standardised rates between 1990 and 2017	2017 age-standardised rates per 100,000	Percentage change in age-standardised rates between 1990 and 2017
High SDI	Andorra	23 (20 to 26)	-35.2% (-46.5% to -21.8%)	854 (738 to 980)	-43.5% (-54.6% to -30.1%)	911 (650 to 1 243)	2.5% (1.1% to 3.9%)	1 765 (1 473 to 2 109)	-26.4% (-35.3% to -16.5%)
High SDI	Australia	30 (27 to 33)	-32.1% (-38.7% to -25.5%)	1 156 (1 033 to 1 284)	-43.2% (-49.4% to -37.1%)	1 514 (1 077 to 2 057)	11.6% (10.0% to 13.2%)	2 670 (2 207 to 3 237)	-21.3% (-27.8% to -15.6%)
High SDI	Austria	29 (27 to 31)	-50.8% (-53.9% to -47.8%)	1 009 (946 to 1 079)	-57.8% (-60.5% to -54.9%)	892 (633 to 1 220)	-9.0% (-10.3% to -7.7%)	1 901 (1 621 to 2 219)	-43.6% (-47.5% to -39.8%)
High SDI	Belgium	38 (36 to 40)	-33.9% (-37.6% to -30.0%)	1 348 (1 267 to 1 431)	-44.6% (-48.2% to -41.2%)	968 (688 to 1 322)	6.6% (4.9% to 8.3%)	2 316 (2 028 to 2 695)	-30.7% (-34.7% to -26.3%)
High SDI	Brunei	44 (41 to 47)	-36.0% (-41.7% to -29.6%)	1 839 (1 704 to 2 003)	-36.6% (-42.1% to -30.0%)	1 031 (725 to 1 411)	-11.9% (-15.0% to -9.0%)	2 871 (2 523 to 3 283)	-29.5% (-34.2% to -24.4%)
High SDI	Canada	33 (31 to 35)	-29.3% (-33.1% to -25.2%)	1 301 (1 225 to 1 375)	-38.3% (-41.9% to -34.4%)	833 (593 to 1 132)	0.2% (-1.1% to 1.5%)	2 134 (1 890 to 2 437)	-27.4% (-31.2% to -24.0%)
High SDI	Croatia	38 (36 to 41)	-49.4% (-52.2% to -46.3%)	1 223 (1 149 to 1 295)	-59.4% (-61.9% to -56.8%)	1 389 (996 to 1 872)	-10.7% (-12.7% to -8.2%)	2 612 (2 219 to 3 098)	-42.8% (-46.3% to -39.3%)
High SDI	Cyprus	28 (25 to 31)	-48.6% (-53.9% to -42.9%)	1 061 (963 to 1 170)	-49.6% (-55.1% to -44.3%)	893 (633 to 1 218)	-8.8% (-10.9% to -6.7%)	1 954 (1 673 to 2 298)	-36.7% (-41.7% to -32.0%)
High SDI	Czech Republic	35 (33 to 37)	-55.9% (-58.5% to -53.1%)	1 280 (1 203 to 1 361)	-54.6% (-57.3% to -51.4%)	2 009 (1 433 to 2 748)	3.3% (1.3% to 5.4%)	3 289 (2 702 to 4 037)	-30.9% (-36.0% to -25.9%)
High SDI	Denmark	25 (23 to 26)	-60.3% (-62.7% to -57.6%)	799 (745 to 855)	-64.2% (-66.7% to -61.6%)	866 (617 to 1 183)	-1.8% (-3.5% to -0.2%)	1 665 (1 404 to 1 995)	-46.5% (-51.2% to -42.1%)
High SDI	Estonia	42 (37 to 48)	-65.9% (-70.3% to -61.1%)	1 771 (1 548 to 2 018)	-69.8% (-73.6% to -65.5%)	1 400 (996 to 1 904)	-18.3% (-20.0% to -16.7%)	3 171 (2 703 to 3 730)	-58.2% (-62.4% to -53.7%)
High SDI	Finland	35 (33 to 38)	-52.4% (-55.3% to -49.0%)	1 282 (1 203 to 1 380)	-58.8% (-61.5% to -55.7%)	1 014 (720 to 1 384)	4.4% (2.9% to 5.9%)	2 296 (1 993 to 2 681)	-43.8% (-47.9% to -39.3%)
High SDI	France	35 (33 to 37)	-51.9% (-54.8% to -48.7%)	1 202 (1 128 to 1 277)	-55.7% (-58.6% to -52.8%)	919 (654 to 1 258)	-5.1% (-6.5% to -3.8%)	2 121 (1 842 to 2 452)	-42.4% (-46.5% to -38.6%)
High SDI	Georgia	52 (49 to 54)	-12.8% (-18.1% to -7.3%)	2 300 (2 187 to 2 407)	-21.0% (-26.3% to -15.6%)	1 034 (741 to 1 398)	-8.1% (-10.4% to -5.7%)	3 334 (3 024 to 3 705)	-17.4% (-21.6% to -13.2%)
High SDI	Germany	26 (24 to 29)	-43.1% (-48.3% to -37.2%)	919 (826 to 1 022)	-52.4% (-57.1% to -47.1%)	869 (615 to 1 192)	2.4% (0.9% to 3.8%)	1 787 (1 521 to 2 109)	-35.6% (-40.7% to -30.5%)
High SDI	Greece	24 (23 to 25)	-39.7% (-43.1% to -36.2%)	998 (938 to 1 062)	-43.2% (-46.9% to -39.5%)	861 (611 to 1 177)	-2.2% (-3.5% to -0.8%)	1 859 (1 594 to 2 175)	-29.6% (-33.7% to -25.4%)
High SDI	Iceland	26 (25 to 27)	-40.9% (-44.0% to -37.3%)	949 (906 to 997)	-48.3% (-51.2% to -45.1%)	881 (627 to 1 204)	3.0% (1.6% to 4.5%)	1 830 (1 565 to 2 160)	-32.0% (-36.5% to -27.5%)
High SDI	Ireland	21 (19 to 22)	-48.6% (-52.3% to -44.8%)	814 (754 to 876)	-52.6% (-56.1% to -49.0%)	860 (610 to 1 169)	4.6% (3.1% to 6.4%)	1 674 (1 417 to 1 979)	-34.1% (-39.1% to -29.6%)
High SDI	Italy	20 (19 to 21)	-52.5% (-55.5% to -49.6%)	686 (640 to 733)	-58.3% (-61.0% to -55.4%)	772 (549 to 1 052)	-11.7% (-13.0% to -10.3%)	1 458 (1 237 to 1 739)	-42.1% (-46.1% to -38.1%)
High SDI	Japan	30 (29 to 31)	-25.5% (-27.6% to -22.8%)	1 136 (1 101 to 1 178)	-30.4% (-32.4% to -27.7%)	1 052 (754 to 1 430)	16.0% (14.5% to 17.7%)	2 188 (1 894 to 2 550)	-13.8% (-17.4% to -10.2%)
High SDI	Latvia	61 (54 to 68)	-52.1% (-57.5% to -46.4%)	2 572 (2 282 to 2 884)	-56.7% (-61.6% to -51.5%)	1 402 (996 to 1 910)	-21.5% (-23.4% to -19.6%)	3 974 (3 443 to 4 553)	-48.6% (-53.3% to -44.1%)
High SDI	Lithuania	75 (71 to 80)	-33.1% (-37.3% to -28.5%)	3 140 (2 945 to 3 369)	-40.2% (-44.2% to -35.9%)	1 482 (1 053 to 2 016)	-9.3% (-11.2% to -7.4%)	4 622 (4 143 to 5 156)	-32.9% (-36.4% to -29.1%)
High SDI	Luxembourg	28 (26 to 31)	-51.4% (-56.1% to -46.6%)	938 (847 to 1 034)	-62.0% (-65.8% to -57.9%)	931 (662 to 1 264)	-10.3% (-11.6% to -8.7%)	1 869 (1 587 to 2 203)	-46.6% (-51.0% to -42.2%)
High SDI	Malta	21 (20 to 22)	-29.1% (-33.1% to -25.0%)	739 (701 to 780)	-32.5% (-36.7% to -27.5%)	919 (654 to 1 253)	8.4% (6.7% to 10.3%)	1 657 (1 384 to 1 993)	-14.6% (-19.3% to -10.4%)
High SDI	Netherlands	26 (25 to 28)	-25.8% (-30.0% to -21.3%)	803 (759 to 855)	-42.1% (-45.3% to -38.1%)	727 (516 to 991)	1.2% (-0.4% to 3.0%)	1 530 (1 306 to 1 796)	-27.3% (-31.4% to -23.5%)
High SDI	New Zealand	32 (31 to 34)	-39.7% (-42.8% to -36.2%)	1 410 (1 339 to 1 491)	-47.0% (-49.8% to -44.1%)	1 860 (1 321 to 2 527)	9.9% (7.7% to 12.3%)	3 270 (2 724 to 3 968)	-24.9% (-29.9% to -20.3%)
High SDI	Norway	29 (28 to 29)	-46.7% (-48.5% to -44.7%)	970 (942 to 1 007)	-55.9% (-57.4% to -54.1%)	1 031 (746 to 1 383)	2.0% (0.8% to 3.3%)	2 001 (1 720 to 2 353)	-37.6% (-41.6% to -33.7%)
High SDI	Poland	41 (39 to 44)	-40.9% (-44.2% to -37.2%)	1 657 (1 563 to 1 760)	-46.0% (-49.3% to -42.5%)	1 708 (1 217 to 2 332)	-8.3% (-11.4% to -5.3%)	3 365 (2 865 to 3 992)	-31.8% (-35.8% to -28.0%)
High SDI	Singapore	15 (14 to 16)	-56.8% (-59.7% to -54.0%)	611 (571 to 650)	-59.1% (-61.8% to -56.5%)	966 (683 to 1 323)	2.9% (0.8% to 4.8%)	1 577 (1 296 to 1 925)	-35.2% (-40.7% to -30.0%)
High SDI	Slovakia	38 (35 to 41)	-48.8% (-52.3% to -44.2%)	1 468 (1 372 to 1 583)	-51.6% (-54.9% to -47.3%)	1 716 (1 227 to 2 349)	-10.5% (-12.4% to -8.6%)	3 184 (2 671 to 3 831)	-35.7% (-39.4% to -31.4%)
High SDI	Slovenia	40 (37 to 42)	-52.3% (-55.5% to -48.9%)	1 318 (1 222 to 1 409)	-59.5% (-62.6% to -56.5%)	2 034 (1 459 to 2 781)	1.4% (-0.0% to 2.8%)	3 353 (2 772 to 4 094)	-36.3% (-41.6% to -31.6%)
High SDI	South Korea	43 (39 to 46)	-48.1% (-52.1% to -44.1%)	1 463 (1 357 to 1 569)	-61.1% (-64.3% to -57.8%)	912 (645 to 1 249)	-24.0% (-26.7% to -20.9%)	2 374 (2 098 to 2 719)	-52.1% (-55.4% to -48.6%)
High SDI	Spain	19 (18 to 20)	-57.5% (-60.0% to -54.7%)	668 (625 to 709)	-67.2% (-69.3% to -65.0%)	836 (595 to 1 140)	-3.6% (-5.0% to -2.1%)	1 504 (1 253 to 1 806)	-48.2% (-52.8% to -43.8%)
High SDI	Sweden	27 (26 to 29)	-38.0% (-41.1% to -34.9%)	950 (899 to 1 005)	-47.0% (-50.0% to -43.9%)	958 (693 to 1 292)	7.3% (5.7% to 8.9%)	1 907 (1 633 to 2 237)	-28.9% (-33.3% to -24.7%)
High SDI	Switzerland	25 (24 to 27)	-58.2% (-61.0% to -55.2%)	804 (751 to 860)	-66.4% (-68.7% to -63.8%)	879 (629 to 1 199)	-21.1% (-22.5% to -19.7%)	1 683 (1 421 to 2 015)	-52.0% (-55.7% to -48.5%)
High SDI	Taiwan (Province of China)	40 (39 to 43)	-54.2% (-56.5% to -51.8%)	1 580 (1 497 to 1 667)	-59.8% (-61.9% to -57.5%)	508 (373 to 669)	-1.9% (-5.0% to 1.1%)	2 088 (1 936 to 2 260)	-53.1% (-55.6% to -50.4%)
High SDI	USA	46 (45 to 47)	-17.2% (-19.6% to -14.9%)	2 024 (1 963 to 2 081)	-26.0% (-28.3% to -23.8%)	811 (589 to 1 093)	-31.2% (-33.2% to -29.5%)	2 836 (2 605 to 3 129)	-27.6% (-29.4% to -25.7%)
High SDI	United Kingdom	21 (21 to 21)	-33.0% (-34.2% to -31.9%)	774 (762 to 787)	-44.3% (-45.4% to -43.3%)	850 (603 to 1 160)	9.8% (8.9% to 10.7%)	1 623 (1 377 to 1 938)	-25.0% (-29.1% to -20.8%)
High-middle SDI	American Samoa	52 (48 to 56)	-17.3% (-25.7% to -8.7%)	2 201 (2 029 to 2 403)	-19.5% (-27.8% to -10.6%)	553 (413 to 720)	18.0% (13.8% to 22.7%)	2 755 (2 516 to 3 007)	-14.0% (-21.6% to -6.1%)
High-middle SDI	Antigua and Barbuda	37 (34 to 40)	-20.2% (-27.1% to -13.2%)	1 698 (1 556 to 1 847)	-15.8% (-24.0% to -7.3%)	406 (294 to 545)	6.0% (1.7% to 10.5%)	2 104 (1 929 to 2 315)	-12.3% (-19.3% to -5.1%)
High-middle SDI	Argentina	47 (43 to 53)	-19.0% (-27.2% to -9.4%)	2 140 (1 917 to 2 391)	-19.2% (-27.6% to -9.2%)	881 (628 to 1 194)	-1.5% (-5.8% to 2.8%)	3 021 (2 676 to 3 424)	-14.7% (-21.4% to -6.9%)
High-middle SDI	Armenia	32 (30 to 33)	-56.1% (-58.3% to -53.8%)	1 311 (1 261 to 1 363)	-62.0% (-64.3% to -59.8%)	1 027 (742 to 1 374)	-30.1% (-33.9% to -26.3%)	2 338 (2 049 to 2 692)	-52.5% (-55.3% to -49.6%)
High-middle SDI	Azerbaijan	31 (28 to 35)	-46.7% (-53.0% to -37.9%)	1 471 (1 311 to 1 686)	-51.6% (-57.7% to -44.0%)	1 049 (756 to 1 410)	-9.0% (-13.2% to -4.4%)	2 520 (2 177 to 2 923)	-39.9% (-45.2% to -33.6%)
High-middle SDI	Bahrain	23 (21 to 26)	-50.6% (-56.2% to -44.3%)	1 014 (922 to 1 126)	-50.4% (-55.5% to -44.4%)	575 (411 to 773)	-13.8% (-19.1% to -8.3%)	1 589 (1 406 to 1 824)	-41.4% (-46.2% to -35.9%)
High-middle SDI	Barbados	37 (34 to 41)	-19.7% (-26.4% to -12.6%)	1 649 (1 512 to 1 795)	-20.9% (-28.0% to -13.4%)	376 (273 to 504)	14.1% (9.5% to 18.5%)	2 025 (1 851 to 2 220)	-16.1% (-22.3% to -9.6%)
High-middle SDI	Belarus	65 (61 to 69)	-29.2% (-33.6% to -24.0%)	2 753 (2 570 to 2 949)	-38.7% (-43.0% to -33.8%)	1 401 (999 to 1 912)	-6.4% (-9.1% to -3.8%)	4 154 (3 705 to 4 682)	-30.7% (-34.5% to -26.4%)
High-middle SDI	Bermuda	24 (22 to 27)	-50.5% (-55.1% to -45.5%)	1 004 (899 to 1 116)	-54.4% (-59.6% to -48.5%)	428 (310 to 568)	6.7% (0.9% to 11.6%)	1 432 (1 267 to 1 607)	-45.0% (-50.2% to -39.5%)
High-middle SDI	Bosnia and Herzegovina	25 (23 to 29)	-35.9% (-44.2% to -18.9%)	1 026 (951 to 1 116)	-38.6% (-45.7% to -25.2%)	1 780 (1 293 to 2 393)	20.5% (13.7% to 30.0%)	2 806 (2 313 to 3 417)	-10.8% (-18.7% to -0.7%)
High-middle SDI	Bulgaria	34 (32 to 37)	-41.4% (-44.9% to -37.8%)	1 488 (1 393 to 1 595)	-45.3% (-48.8% to -41.6%)	1 573 (1 111 to 2 152)	-9.6% (-11.8% to -7.4%)	3 061 (2 595 to 3 640)	-31.3% (-35.3% to -27.7%)
High-middle SDI	Chile	40 (36 to 45)	-48.8% (-54.4% to -42.4%)	1 688 (1 494 to 1 903)	-53.3% (-58.7% to -47.0%)	740 (524 to 1 006)	-16.3% (-20.2% to -12.0%)	2 428 (2 125 to 2 778)	-46.1% (-51.1% to -40.9%)
High-middle SDI	China	46 (43 to 48)	-44.3% (-48.9% to -41.1%)	1 893 (1 798 to 1 963)	-55.8% (-58.8% to -53.4%)	580 (430 to 758)	21.2% (14.9% to 27.9%)	2 473 (2 298 to 2 679)	-48.1% (-51.8% to -44.6%)
High-middle SDI	Georgia	52 (49 to 54)	-12.8% (-18.1% to -7.3%)	2 300 (2 187 to 2 407)	-21.0% (-26.3% to -15.6%)	1 034 (741 to 1 398)	-8.1% (-10.4% to -5.7%)	3 334 (3 024 to 3 705)	-17.4% (-21.6% to -13.2%)
High-middle SDI	Greenland	104 (98 to 112)	-51.2% (-55.0% to -46.6%)	4 474 (4 153 to 4 799)	-58.4% (-61.9% to -54.5%)	948 (682 to 1 281)	-39.8% (-41.8% to -38.0%)	5 422 (5 006 to 5 864)	-56.0% (-59.2% to -52.6%)
High-middle SDI	Guam	57 (53 to 62)	1.5% (-8.5% to 12.5%)	2 655 (2 441 to 2 881)	4.0% (-6.0% to 15.2%)	548 (400 to 726)	30.5% (26.7% to 34.1%)	3 203 (2 938 to 3 479)	7.8% (-1.2% to 17.7%)
High-middle SDI	Hungary	39 (37 to 41)	-63.0% (-65.0% to -60.9%)	1 334 (1 260 to 1 414)	-64.4% (-66.4% to -62.2%)	1 636 (1 166 to 2 240)	-15.0% (-17.5% to -12.5%)	2 970 (2 484 to 3 567)	-47.6% (-51.7% to -43.5%)
High-middle SDI	Iran	53 (52 to 54)	-70.1% (-71.9% to -68.7%)	2 512 (2 464 to 2 611)	-72.5% (-73.4% to -71.3%)	732 (543 to 962)	-36.4% (-42.1% to -30.7%)	3 244 (3 051 to 3 499)	-68.4% (-69.9% to -66.8%)
High-middle SDI	Israel	23 (21 to 24)	-44.7% (-48.3% to -37.4%)	831 (782 to 887)	-49.3% (-52.7% to -44.3%)	882 (630 to 1 194)	6.9% (4.2% to 11.5%)	1 713 (1 454 to 2 022)	-30.5% (-35.2% to -25.3%)
High-middle SDI	Kazakhstan	74 (70 to 79)	-26.5% (-30.4% to -22.2%)	3 501 (3 295 to 3 757)	-31.4% (-35.4% to -26.3%)	1 074 (766 to 1 453)	-13.8% (-17.4% to -10.5%)	4 574 (4 192 to 5 017)	-27.9% (-31.6% to -23.8%)
High-middle SDI	Kuwait	25 (23 to 26)	-78.7% (-80.1% to -77.2%)	1 073 (1 004 to 1 146)	-82.5% (-83.6% to -81.3%)	626 (449 to 843)	-16.7% (-20.7% to -13.0%)	1 700 (1 517 to 1 939)	-75.3% (-77.4% to -72.7%)
High-middle SDI	Lebanon	33 (29 to 37)	-67.0% (-71.8% to -62.0%)	1 447 (1 282 to 1 603)	-71.2% (-75.1% to -67.6%)	803 (585 to 1 061)	-39.5% (-47.3% to -31.6%)	2 249 (1 985 to 2 553)	-64.6% (-68.2% to -61.2%)
High-middle SDI	Libya	85 (71 to 99)	24.8% (3.1% to 48.4%)	4 132 (3 534 to 4 734)	28.3% (7.4% to 51.4%)	855 (629 to 1 127)	16.3% (2.8% to 40.1%)	4 987 (4 343 to 5 644)	26.1% (8.9% to 43.7%)
High-middle SDI	Macedonia	24 (22 to 26)	-38.9% (-44.2% to -33.7%)	997 (932 to 1 070)	-43.4% (-48.3% to -38.6%)	1 529 (1 088 to 2 101)	0.9% (-2.7% to 4.4%)	2 526 (2 065 to 3 095)	-22.9% (-28.4% to -17.9%)
High-middle SDI	Malaysia	52 (47 to 57)	-25.8% (-36.6% to -16.9%)	2 015 (1 844 to 2 215)	-27.8% (-36.6% to -19.4%)	394 (284 to 524)	4.1% (-1.9% to 10.4%)	2 409 (2 200 to 2 644)	-24.0% (-32.2% to -16.5%)
High-middle SDI	Mauritius	38 (36 to 41)	-23.4% (-29.1% to -17.8%)	1 746 (1 617 to 1 868)	-23.1% (-28.9% to -17.3%)	316 (230 to 417)	17.7% (12.9% to 22.4%)	2 062 (1 915 to 2 214)	-18.8% (-24.1% to -13.4%)

SDI Quintile	Location	Deaths (95% UI)		YLLs (95% UI)		YLDs (95% UI)		DALYs (95% UI)	
		2017 age-standardised rates per 100,000	Percentage change in age-standardised rates between 1990 and 2017	2017 age-standardised rates per 100,000	Percentage change in age-standardised rates between 1990 and 2017	2017 age-standardised rates per 100,000	Percentage change in age-standardised rates between 1990 and 2017	2017 age-standardised rates per 100,000	Percentage change in age-standardised rates between 1990 and 2017
High-middle SDI	Montenegro	33 (30 to 36)	-31.3% (-37.5% to -24.4%)	1 341 (1 221 to 1 468)	-40.0% (-45.7% to -33.6%)	1 593 (1 133 to 2 174)	2.0% (0.2% to 3.7%)	2 934 (2 443 to 3 547)	-22.7% (-28.0% to -17.9%)
High-middle SDI	Northern Mariana Islands	51 (45 to 56)	-20.3% (-32.3% to -6.1%)	2 110 (1 852 to 2 355)	-19.2% (-32.7% to -2.9%)	493 (359 to 657)	4.9% (1.6% to 8.4%)	2 603 (2 335 to 2 900)	-15.6% (-26.9% to -1.7%)
High-middle SDI	Oman	61 (50 to 72)	-50.4% (-60.5% to -37.1%)	2 601 (2 165 to 3 065)	-54.3% (-63.5% to -41.9%)	611 (431 to 830)	-20.3% (-25.2% to -15.3%)	3 212 (2 733 to 3 736)	-50.3% (-58.8% to -38.8%)
High-middle SDI	Portugal	25 (24 to 27)	-64.0% (-66.4% to -61.6%)	930 (863 to 997)	-71.7% (-73.8% to -69.5%)	729 (517 to 998)	-27.8% (-29.7% to -26.0%)	1 659 (1 436 to 1 910)	-61.4% (-64.6% to -58.4%)
High-middle SDI	Puerto Rico	105 (102 to 109)	50.1% (44.8% to 55.7%)	5 175 (5 011 to 5 336)	57.8% (52.0% to 63.9%)	478 (341 to 640)	31.4% (27.3% to 35.9%)	5 653 (5 442 to 5 868)	55.1% (49.9% to 60.6%)
High-middle SDI	Qatar	43 (35 to 51)	-37.4% (-50.1% to -20.5%)	1 785 (1 469 to 2 136)	-42.1% (-53.9% to -26.8%)	670 (475 to 909)	-16.9% (-20.5% to -13.0%)	2 454 (2 056 to 2 883)	-36.9% (-46.9% to -24.7%)
High-middle SDI	Romania	40 (38 to 42)	-43.7% (-46.7% to -40.7%)	1 721 (1 630 to 1 823)	-50.9% (-53.6% to -48.1%)	1 596 (1 131 to 2 193)	-23.0% (-26.1% to -20.0%)	3 317 (2 838 to 3 909)	-40.6% (-43.8% to -37.5%)
High-middle SDI	Russian Federation	87 (86 to 89)	-24.0% (-25.1% to -23.1%)	3 987 (3 925 to 4 056)	-28.2% (-29.1% to -27.4%)	1 444 (1 026 to 1 962)	-9.2% (-11.9% to -6.7%)	5 431 (5 008 to 5 946)	-24.0% (-25.6% to -22.3%)
High-middle SDI	Saudi Arabia	72 (61 to 83)	-30.3% (-46.7% to -12.7%)	2 989 (2 516 to 3 461)	-35.4% (-49.0% to -19.8%)	675 (481 to 915)	-24.9% (-29.6% to -20.0%)	3 664 (3 136 to 4 208)	-33.7% (-45.5% to -20.8%)
High-middle SDI	Serbia	33 (31 to 35)	-43.1% (-47.2% to -38.7%)	1 213 (1 132 to 1 309)	-54.5% (-58.0% to -50.2%)	1 566 (1 118 to 2 133)	4.5% (1.8% to 7.6%)	2 779 (2 324 to 3 356)	-33.2% (-38.4% to -27.6%)
High-middle SDI	The Bahamas	71 (65 to 77)	-6.0% (-14.2% to 3.0%)	3 382 (3 085 to 3 681)	-2.5% (-11.7% to 7.2%)	407 (296 to 543)	4.1% (0.2% to 7.6%)	3 789 (3 469 to 4 130)	-1.9% (-10.2% to 7.0%)
High-middle SDI	Turkey	31 (28 to 34)	-38.0% (-45.7% to -30.5%)	1 432 (1 318 to 1 556)	-43.5% (-50.0% to -35.8%)	552 (395 to 740)	-24.3% (-30.1% to -17.9%)	1 984 (1 785 to 2 197)	-39.2% (-45.1% to -32.6%)
High-middle SDI	Ukraine	80 (76 to 85)	-11.6% (-16.7% to -6.0%)	3 888 (3 681 to 4 115)	-12.7% (-17.9% to -6.9%)	1 371 (971 to 1 872)	-7.5% (-10.1% to -4.8%)	5 259 (4 810 to 5 777)	-11.4% (-15.3% to -7.0%)
High-middle SDI	United Arab Emirates	78 (63 to 95)	-18.7% (-40.4% to 8.2%)	3 005 (2 432 to 3 638)	-21.7% (-40.9% to 0.8%)	707 (510 to 956)	-25.6% (-29.6% to -21.2%)	3 712 (3 095 to 4 399)	-22.5% (-38.0% to -5.2%)
High-middle SDI	Uruguay	59 (53 to 66)	-6.7% (-16.7% to 3.1%)	2 553 (2 276 to 2 833)	-12.6% (-22.3% to -2.5%)	877 (624 to 1 192)	-1.6% (-5.2% to 1.8%)	3 430 (3 064 to 3 855)	-10.0% (-17.7% to -2.3%)
High-middle SDI	Virgin Islands	69 (63 to 77)	-4.6% (-14.8% to 7.4%)	2 874 (2 630 to 3 205)	-9.4% (-18.5% to 2.6%)	413 (295 to 557)	7.7% (2.5% to 13.6%)	3 287 (3 018 to 3 667)	-7.6% (-15.9% to 3.2%)
Middle SDI	Albania	26 (22 to 31)	-37.1% (-47.5% to -25.8%)	1 236 (1 037 to 1 454)	-41.7% (-51.5% to -31.2%)	1 547 (1 101 to 2 124)	-4.6% (-8.8% to -0.4%)	2 783 (2 272 to 3 380)	-25.6% (-33.0% to -18.7%)
Middle SDI	Algeria	36 (31 to 47)	-44.2% (-49.2% to -38.7%)	1 731 (1 483 to 2 172)	-48.2% (-53.4% to -42.2%)	624 (455 to 820)	-18.9% (-23.4% to -13.7%)	2 355 (2 039 to 2 823)	-42.7% (-47.4% to -37.7%)
Middle SDI	Botswana	51 (46 to 58)	-29.0% (-42.6% to -13.5%)	2 185 (1 929 to 2 487)	-30.6% (-45.0% to -13.3%)	564 (417 to 741)	-10.9% (-14.5% to -7.2%)	2 749 (2 449 to 3 106)	-27.3% (-40.3% to -12.5%)
Middle SDI	Brazil	74 (73 to 75)	-24.0% (-25.6% to -22.2%)	3 573 (3 516 to 3 625)	-25.4% (-27.1% to -23.0%)	512 (375 to 675)	16.1% (13.4% to 18.8%)	4 085 (3 936 to 4 247)	-21.9% (-23.9% to -19.5%)
Middle SDI	Colombia	61 (53 to 68)	-55.8% (-60.9% to -50.2%)	3 114 (2 753 to 3 501)	-56.0% (-61.1% to -50.4%)	372 (277 to 486)	-29.7% (-34.0% to -25.1%)	3 486 (3 101 to 3 876)	-54.1% (-59.2% to -49.0%)
Middle SDI	Costa Rica	51 (47 to 54)	-14.5% (-20.4% to -8.1%)	2 130 (1 987 to 2 280)	-8.5% (-14.9% to -1.4%)	337 (242 to 451)	4.5% (-0.1% to 9.1%)	2 468 (2 299 to 2 660)	-6.9% (-12.6% to -0.6%)
Middle SDI	Cuba	48 (43 to 53)	-39.6% (-45.3% to -32.9%)	1 641 (1 473 to 1 846)	-50.3% (-55.3% to -44.2%)	409 (292 to 553)	8.5% (4.5% to 12.7%)	2 051 (1 844 to 2 267)	-44.2% (-49.1% to -38.7%)
Middle SDI	Dominica	56 (52 to 60)	-1.7% (-9.2% to 6.6%)	2 732 (2 516 to 2 987)	4.1% (-5.1% to 14.5%)	421 (308 to 558)	20.4% (15.5% to 25.1%)	3 152 (2 913 to 3 432)	6.0% (-2.3% to 15.1%)
Middle SDI	Ecuador	73 (66 to 80)	-14.1% (-22.0% to -4.9%)	3 393 (3 070 to 3 759)	-13.5% (-21.9% to -3.7%)	477 (353 to 628)	-16.0% (-22.0% to -9.8%)	3 869 (3 532 to 4 258)	-13.8% (-21.3% to -5.3%)
Middle SDI	Equatorial Guinea	56 (40 to 78)	-61.9% (-73.0% to -47.3%)	2 312 (1 604 to 3 229)	-68.7% (-78.3% to -54.6%)	615 (459 to 801)	-18.7% (-23.4% to -13.3%)	2 927 (2 201 to 3 904)	-64.1% (-73.4% to -50.8%)
Middle SDI	Fiji	49 (44 to 55)	-8.0% (-21.9% to 7.7%)	2 336 (2 068 to 2 648)	-6.6% (-20.8% to 10.1%)	526 (389 to 679)	27.0% (23.1% to 30.8%)	2 862 (2 562 to 3 210)	-1.8% (-15.1% to 13.2%)
Middle SDI	Gabon	72 (61 to 84)	-30.0% (-41.7% to -15.7%)	3 018 (2 442 to 3 602)	-34.9% (-47.5% to -20.3%)	730 (547 to 947)	-19.0% (-21.1% to -16.8%)	3 748 (3 161 to 4 374)	-32.3% (-43.0% to -20.3%)
Middle SDI	Grenada	45 (42 to 48)	-32.5% (-37.0% to -27.3%)	1 902 (1 774 to 2 044)	-35.0% (-40.2% to -29.0%)	439 (321 to 582)	7.7% (3.2% to 12.0%)	2 341 (2 160 to 2 535)	-29.8% (-35.0% to -24.3%)
Middle SDI	Indonesia	39 (37 to 42)	-49.5% (-54.1% to -43.9%)	1 560 (1 473 to 1 648)	-56.1% (-59.4% to -51.2%)	359 (271 to 458)	-3.8% (-6.3% to -1.1%)	1 919 (1 787 to 2 050)	-51.1% (-54.5% to -46.2%)
Middle SDI	Jamaica	56 (47 to 65)	89.2% (58.4% to 123.0%)	2 616 (2 209 to 3 055)	94.5% (62.2% to 130.8%)	449 (324 to 601)	21.9% (16.7% to 26.7%)	3 065 (2 630 to 3 529)	78.9% (53.7% to 107.7%)
Middle SDI	Jordan	29 (26 to 32)	-51.5% (-57.9% to -43.4%)	1 387 (1 247 to 1 542)	-54.3% (-60.8% to -46.2%)	494 (352 to 662)	-25.4% (-29.5% to -20.9%)	1 881 (1 665 to 2 112)	-49.1% (-54.9% to -41.8%)
Middle SDI	Maldives	26 (24 to 33)	-65.9% (-69.7% to -58.0%)	958 (855 to 1 225)	-72.5% (-75.9% to -65.0%)	324 (237 to 426)	-8.2% (-14.6% to -1.3%)	1 282 (1 138 to 1 573)	-66.6% (-70.6% to -58.9%)
Middle SDI	Mexico	74 (73 to 75)	-19.4% (-21.1% to -18.1%)	3 535 (3 486 to 3 588)	-16.1% (-17.8% to -14.3%)	437 (321 to 579)	-8.7% (-11.2% to -6.6%)	3 971 (3 842 to 4 120)	-15.4% (-16.9% to -13.7%)
Middle SDI	Moldova	59 (56 to 61)	-42.4% (-45.1% to -39.6%)	2 598 (2 475 to 2 735)	-49.6% (-52.7% to -46.0%)	1 291 (927 to 1 754)	-19.1% (-21.8% to -16.5%)	3 889 (3 518 to 4 320)	-42.4% (-45.3% to -39.3%)
Middle SDI	Mongolia	72 (65 to 81)	-21.7% (-31.9% to -11.1%)	3 610 (3 229 to 4 037)	-21.0% (-30.7% to -10.4%)	1 293 (938 to 1 723)	-2.3% (-6.4% to 2.3%)	4 903 (4 355 to 5 520)	-16.8% (-25.0% to -8.5%)
Middle SDI	Namibia	72 (59 to 87)	-37.4% (-49.0% to -23.8%)	3 205 (2 551 to 3 994)	-38.0% (-50.6% to -22.8%)	752 (563 to 963)	-38.7% (-47.2% to -31.3%)	3 957 (3 288 to 4 766)	-38.1% (-48.4% to -25.9%)
Middle SDI	Panama	47 (44 to 51)	-25.7% (-31.4% to -19.8%)	2 319 (2 119 to 2 519)	-23.3% (-29.8% to -16.6%)	343 (250 to 455)	-3.7% (-9.1% to 2.0%)	2 663 (2 451 to 2 883)	-21.2% (-27.2% to -15.1%)
Middle SDI	Paraguay	58 (48 to 69)	12.5% (-7.9% to 37.3%)	2 572 (2 138 to 3 096)	5.8% (-14.2% to 29.7%)	460 (334 to 612)	1.2% (-3.2% to 5.5%)	3 032 (2 592 to 3 579)	5.1% (-11.8% to 25.2%)
Middle SDI	Peru	41 (35 to 47)	-55.5% (-62.2% to -48.0%)	1 913 (1 627 to 2 215)	-64.3% (-70.3% to -57.7%)	442 (325 to 581)	-19.9% (-26.3% to -13.1%)	2 354 (2 038 to 2 689)	-60.1% (-65.7% to -53.8%)
Middle SDI	Philippines	56 (49 to 64)	-20.5% (-30.4% to -8.9%)	2 536 (2 238 to 2 891)	-23.5% (-33.4% to -12.4%)	359 (268 to 463)	17.6% (13.3% to 22.0%)	2 895 (2 575 to 3 260)	-20.1% (-29.2% to -9.8%)
Middle SDI	Saint Lucia	57 (53 to 61)	-20.3% (-26.0% to -14.1%)	2 591 (2 409 to 2 782)	-17.9% (-24.1% to -10.9%)	410 (299 to 546)	4.7% (-0.2% to 9.4%)	3 001 (2 798 to 3 222)	-15.4% (-21.2% to -8.9%)
Middle SDI	Saint Vincent and the Grenadines	63 (59 to 67)	4.3% (-3.1% to 12.0%)	2 917 (2 731 to 3 107)	7.0% (-1.5% to 15.7%)	455 (334 to 601)	26.9% (22.4% to 31.0%)	3 372 (3 156 to 3 618)	9.3% (1.9% to 17.0%)
Middle SDI	Seychelles	55 (52 to 59)	-19.9% (-25.7% to -13.1%)	2 332 (2 187 to 2 479)	-18.7% (-25.3% to -12.3%)	362 (264 to 481)	14.4% (8.6% to 20.5%)	2 694 (2 515 to 2 883)	-15.4% (-21.4% to -9.5%)
Middle SDI	South Africa	84 (79 to 91)	-45.5% (-50.3% to -41.0%)	3 965 (3 648 to 4 299)	-50.6% (-54.4% to -46.5%)	612 (452 to 808)	-27.7% (-30.1% to -25.1%)	4 577 (4 219 to 4 978)	-48.4% (-52.1% to -44.6%)
Middle SDI	Sri Lanka	62 (52 to 72)	-55.2% (-62.1% to -47.5%)	2 302 (1 900 to 2 728)	-62.4% (-68.9% to -55.6%)	548 (409 to 715)	25.4% (14.2% to 41.8%)	2 850 (2 430 to 3 327)	-56.5% (-62.6% to -49.9%)
Middle SDI	Suriname	79 (71 to 88)	-17.4% (-27.2% to -6.9%)	3 486 (3 107 to 3 886)	-22.1% (-31.3% to -11.9%)	468 (344 to 617)	2.6% (-2.0% to 7.5%)	3 955 (3 581 to 4 377)	-19.8% (-28.4% to -10.3%)
Middle SDI	Syria	271 (267 to 276)	487.5% (421.2% to 563.2%)	14 915 (14 744 to 15 112)	602.2% (530.0% to 696.4%)	1 426 (1 029 to 1 949)	122.6% (87.8% to 182.6%)	16 341 (15 893 to 16 858)	491.0% (437.0% to 556.7%)
Middle SDI	Thailand	61 (55 to 67)	-38.5% (-46.5% to -30.3%)	2 973 (2 675 to 3 267)	-38.8% (-46.7% to -31.0%)	398 (289 to 527)	-4.5% (-9.7% to 1.2%)	3 371 (3 026 to 3 703)	-36.1% (-43.5% to -28.7%)
Middle SDI	Tonga	48 (42 to 54)	-16.1% (-29.1% to -2.0%)	2 093 (1 833 to 2 370)	-14.5% (-28.2% to 0.2%)	528 (392 to 682)	24.8% (20.1% to 29.8%)	2 621 (2 330 to 2 940)	-8.7% (-20.7% to 4.5%)
Middle SDI	Trinidad and Tobago	63 (51 to 76)	-7.0% (-23.8% to 12.2%)	3 073 (2 504 to 3 725)	-3.6% (-21.3% to 16.4%)	421 (306 to 561)	11.3% (6.4% to 16.1%)	3 494 (2 904 to 4 148)	-2.0% (-18.0% to 15.7%)
Middle SDI	Tunisia	48 (40 to 59)	-39.4% (-51.5% to -25.1%)	1 983 (1 608 to 2 380)	-47.3% (-57.3% to -36.0%)	561 (402 to 755)	-22.0% (-26.9% to -16.8%)	2 544 (2 121 to 2 989)	-43.2% (-51.9% to -33.6%)
Middle SDI	Turkmenistan	36 (33 to 39)	-44.8% (-50.0% to -39.0%)	1 811 (1 650 to 2 006)	-49.4% (-54.9% to -42.8%)	1 019 (735 to 1 371)	-13.4% (-17.7% to -8.8%)	2 829 (2 497 to 3 229)	-40.5% (-45.6% to -35.1%)
Middle SDI	Uzbekistan	42 (37 to 46)	-33.0% (-40.5% to -25.0%)	2 032 (1 811 to 2 267)	-38.4% (-45.5% to -31.0%)	970 (696 to 1 308)	-9.1% (-12.6% to -5.4%)	3 002 (2 641 to 3 406)	-31.2% (-37.4% to -25.5%)
Middle SDI	Venezuela	95 (81 to 111)	12.2% (-5.0% to 31.3%)	5 025 (4 242 to 5 890)	20.7% (1.8% to 41.1%)	440 (323 to 581)	2.9% (-2.3% to 9.0%)	5 466 (4 708 to 6 319)	19.0% (2.1% to 37.3%)
Middle SDI	Vietnam	68 (60 to 75)	-25.1% (-36.0% to -13.0%)	2 559 (2 220 to 2 820)	-34.6% (-44.9% to -23.1%)	374 (275 to 494)	11.6% (5.0% to 17.9%)	2 933 (2 578 to 3 225)	-30.9% (-41.0% to -20.3%)
Low-middle SDI	Angola	75 (65 to 87)	-55.6% (-63.9% to -38.1%)	3 278 (2 803 to 3 849)	-63.6% (-71.2% to -45.6%)	955 (718 to 1 212)	-26.2% (-30.6% to -21.8%)	4 234 (3 689 to 4 901)	-58.9% (-66.3% to -42.6%)
Low-middle SDI	Belize	83 (79 to 87)	7.6% (-0.2% to 15.4%)	3 910 (3 710 to 4 119)	6.5% (-1.6% to 15.9%)	494 (367 to 652)	25.3% (20.8% to 29.9%)	4 404 (4 159 to 4 646)	8.3% (0.8% to 16.7%)
Low-middle SDI	Bhutan	50 (40 to 58)	-46.0% (-57.1% to -34.4%)	1 761 (1 378 to 2 086)	-55.5% (-66.4% to -44.0%)	659 (496 to 859)	-12.4% (-16.6% to -7.7%)	2 420 (1 958 to 2 811)	-48.6% (-58.9% to -37.9%)
Low-middle SDI	Bolivia	60 (47 to 72)	-51.1% (-62.7% to -39.4%)	2 580 (1 862 to 3 073)	-58.7% (-70.9% to -47.2%)	444 (326 to 584)	-14.5% (-18.8% to -9.8%)	3 024 (2 301 to 3 564)	-55.3% (-66.8% to -44.2%)
Low-middle SDI	Cambodia	98 (81 to 114)	-40.6% (-48.3% to -31.3%)	3 701 (3 137 to 4 333)	-49.6% (-57.7% to -40.0%)	628 (469 to 812)	-32.9% (-42.1% to -23.9%)	4 329 (3 741 to 5 012)	-47.7% (-54.9% to -39.0%)
Low-middle SDI	Cameroon	79 (65 to 93)	-20.3% (-33.6% to -5.5%)	2 850 (2 325 to 3 403)	-25.4% (-39.3% to -10.2%)	614 (457 to 791)	-15.0% (-17.5% to -12.6%)	3 464 (2 885 to 4 059)	-23.8% (-35.8% to -11.0%)
Low-middle SDI	Cape Verde	58 (53 to 63)	-0.2% (-12.8% to 12.4%)	2 542 (2 293 to 2 810)	-7.3% (-19.7% to 5.5%)	522 (385 to 689)	-9.6% (-13.5% to -5.4%)	3 064 (2 753 to 3 383)	-7.7% (-18.0% to 2.8%)
Low-middle SDI	Congo (Brazzaville)	77 (65 to 92)	-37.4% (-49.4% to -22.5%)	3 295 (2 672 to 3 994)	-43.4% (-55.4% to -27.0%)	850 (639 to 1 089)	10.7% (3.2% to 21.4%)	4 146 (3 471 to 4 893)	-37.1% (-48.4% to -22.4%)
Low-middle SDI	Djibouti	69 (52 to 94)	-32.9% (-50.1% to -9.5%)	2 416 (1 758 to 3 416)	-42.5% (-58.0% to -15.8%)	857 (642 to 1 103)	-14.6% (-17.3% to -11.5%)	3 273 (2 583 to 4 275)	-37.1% (-50.4% to -15.3%)

SDI Quintile	Location	Deaths (95% UI)		YLLs (95% UI)		YLDs (95% UI)		DALYs (95% UI)	
		2017 age-standardised rates per 100,000	Percentage change in age-standardised rates between 1990 and 2017	2017 age-standardised rates per 100,000	Percentage change in age-standardised rates between 1990 and 2017	2017 age-standardised rates per 100,000	Percentage change in age-standardised rates between 1990 and 2017	2017 age-standardised rates per 100,000	Percentage change in age-standardised rates between 1990 and 2017
Low-middle SDI	Dominican Republic	80 (69 to 92)	19.8% (-2.8% to 42.5%)	3 744 (3 221 to 4 283)	10.1% (-10.5% to 30.9%)	464 (337 to 622)	8.9% (1.5% to 17.1%)	4 208 (3 681 to 4 764)	10.0% (-8.7% to 28.2%)
Low-middle SDI	Egypt	54 (44 to 64)	-32.0% (-43.2% to -21.1%)	2 473 (2 109 to 2 828)	-39.9% (-47.7% to -29.0%)	572 (416 to 763)	-17.7% (-23.2% to -11.7%)	3 045 (2 660 to 3 451)	-36.7% (-43.5% to -27.3%)
Low-middle SDI	El Salvador	106 (87 to 125)	-31.7% (-43.7% to -19.0%)	4 924 (4 032 to 5 883)	-35.7% (-47.7% to -23.5%)	560 (415 to 726)	-48.7% (-55.7% to -41.1%)	5 484 (4 572 to 6 440)	-37.4% (-47.9% to -26.3%)
Low-middle SDI	Federated States of Micronesia	73 (52 to 89)	-17.7% (-41.9% to 4.8%)	3 282 (2 157 to 4 160)	-20.4% (-48.0% to 3.3%)	574 (426 to 740)	24.5% (19.8% to 29.1%)	3 856 (2 759 to 4 742)	-15.9% (-41.0% to 6.1%)
Low-middle SDI	Ghana	76 (67 to 85)	-3.0% (-20.1% to 15.0%)	2 646 (2 264 to 3 056)	-11.8% (-26.7% to 5.8%)	606 (453 to 785)	-4.0% (-7.3% to -0.6%)	3 252 (2 843 to 3 725)	-10.4% (-23.1% to 4.1%)
Low-middle SDI	Guatemala	92 (82 to 102)	-27.9% (-35.3% to -19.3%)	4 351 (3 894 to 4 872)	-29.5% (-36.7% to -20.8%)	477 (355 to 616)	-35.7% (-43.4% to -28.2%)	4 828 (4 351 to 5 366)	-30.2% (-37.0% to -22.1%)
Low-middle SDI	Guyana	95 (84 to 107)	-2.2% (-13.5% to 10.3%)	4 246 (3 738 to 4 780)	-1.8% (-13.7% to 11.4%)	470 (345 to 620)	11.3% (6.8% to 15.9%)	4 716 (4 231 to 5 247)	-0.7% (-11.5% to 11.6%)
Low-middle SDI	Honduras	84 (68 to 103)	-29.0% (-44.3% to -10.2%)	3 864 (3 095 to 4 647)	-36.1% (-50.5% to -19.2%)	478 (361 to 612)	14.8% (7.3% to 24.1%)	4 342 (3 549 to 5 175)	-32.8% (-46.3% to -16.9%)
Low-middle SDI	India	85 (78 to 90)	-23.3% (-29.6% to -15.8%)	3 070 (2 794 to 3 231)	-34.0% (-38.2% to -28.5%)	657 (488 to 855)	2.8% (-0.2% to 6.0%)	3 726 (3 396 to 3 990)	-29.5% (-33.7% to -24.2%)
Low-middle SDI	Iraq	115 (112 to 118)	14.0% (0.4% to 36.2%)	6 311 (6 151 to 6 472)	22.7% (8.8% to 48.3%)	1 552 (1 125 to 2 064)	-30.1% (-34.0% to -26.0%)	7 863 (7 434 to 8 416)	6.8% (-2.8% to 21.6%)
Low-middle SDI	Kenya	76 (69 to 84)	-11.4% (-26.3% to -1.2%)	2 568 (2 332 to 2 948)	-11.9% (-23.0% to -2.3%)	788 (588 to 1 033)	6.6% (5.1% to 8.3%)	3 356 (3 042 to 3 800)	-8.1% (-18.1% to -0.2%)
Low-middle SDI	Kyrgyzstan	47 (45 to 49)	-52.7% (-55.6% to -49.3%)	2 199 (2 094 to 2 325)	-56.0% (-59.0% to -52.5%)	978 (708 to 1 311)	-20.2% (-22.4% to -17.9%)	3 177 (2 895 to 3 530)	-48.9% (-52.1% to -45.6%)
Low-middle SDI	Laos	69 (54 to 80)	-49.2% (-60.3% to -37.7%)	3 203 (2 427 to 3 739)	-54.8% (-65.8% to -44.3%)	421 (313 to 544)	-0.3% (-4.2% to 3.8%)	3 624 (2 830 to 4 185)	-51.7% (-62.3% to -41.1%)
Low-middle SDI	Lesotho	156 (126 to 184)	25.8% (-1.5% to 56.5%)	7 282 (5 784 to 8 713)	30.3% (0.9% to 63.8%)	669 (498 to 864)	6.7% (3.7% to 10.0%)	7 951 (6 425 to 9 407)	28.0% (1.4% to 57.4%)
Low-middle SDI	Marshall Islands	94 (80 to 109)	-12.2% (-24.7% to 2.1%)	4 279 (3 618 to 5 057)	-13.9% (-27.0% to 1.0%)	559 (415 to 724)	29.1% (24.8% to 33.1%)	4 838 (4 141 to 5 637)	-10.5% (-22.9% to 3.2%)
Low-middle SDI	Mauritania	62 (54 to 72)	-35.8% (-45.6% to -22.9%)	2 276 (1 949 to 2 680)	-41.2% (-50.9% to -25.8%)	560 (415 to 725)	-18.8% (-21.5% to -15.9%)	2 836 (2 465 to 3 307)	-37.8% (-46.3% to -24.6%)
Low-middle SDI	Morocco	46 (37 to 60)	-36.3% (-47.8% to -24.0%)	2 144 (1 729 to 2 782)	-42.8% (-53.5% to -31.3%)	566 (415 to 753)	-17.3% (-21.8% to -12.4%)	2 710 (2 246 to 3 374)	-38.9% (-48.1% to -29.1%)
Low-middle SDI	Myanmar	77 (68 to 86)	-32.7% (-43.2% to -20.9%)	3 393 (3 002 to 3 837)	-39.3% (-49.8% to -27.3%)	524 (398 to 668)	29.2% (18.6% to 40.8%)	3 917 (3 487 to 4 375)	-34.7% (-45.2% to -22.8%)
Low-middle SDI	Nicaragua	38 (34 to 45)	-46.7% (-53.7% to -37.4%)	1 701 (1 496 to 1 993)	-52.9% (-59.0% to -44.7%)	537 (395 to 701)	-44.2% (-51.1% to -36.9%)	2 238 (1 986 to 2 550)	-51.1% (-56.4% to -44.4%)
Low-middle SDI	Nigeria	55 (45 to 71)	-20.4% (-35.8% to 2.1%)	2 229 (1 801 to 2 795)	-26.1% (-38.9% to -6.1%)	565 (422 to 733)	-10.3% (-13.4% to -7.0%)	2 794 (2 342 to 3 383)	-23.4% (-34.2% to -6.6%)
Low-middle SDI	North Korea	61 (50 to 73)	14.4% (-10.6% to 40.9%)	3 036 (2 462 to 3 645)	14.9% (-11.2% to 44.0%)	525 (395 to 681)	36.3% (31.5% to 40.9%)	3 560 (2 950 to 4 197)	17.6% (-5.7% to 42.7%)
Low-middle SDI	Pakistan	67 (53 to 80)	0.3% (-16.8% to 22.8%)	2 940 (2 324 to 3 513)	-9.5% (-25.5% to 12.2%)	716 (538 to 919)	28.6% (24.3% to 33.1%)	3 655 (3 034 to 4 239)	-3.9% (-18.0% to 15.0%)
Low-middle SDI	Palestine	31 (27 to 34)	-55.3% (-61.8% to -49.6%)	1 455 (1 242 to 1 607)	-60.6% (-66.5% to -54.8%)	1 254 (916 to 1 672)	2.5% (-3.6% to 8.7%)	2 709 (2 321 to 3 152)	-44.9% (-51.0% to -38.9%)
Low-middle SDI	Samoa	47 (39 to 57)	-25.8% (-39.8% to -10.5%)	1 949 (1 539 to 2 447)	-33.3% (-48.0% to -16.7%)	616 (464 to 790)	40.5% (35.3% to 45.9%)	2 565 (2 099 to 3 063)	-23.7% (-37.9% to -8.9%)
Low-middle SDI	Sao Tome and Principe	52 (43 to 61)	-3.2% (-20.7% to 15.6%)	2 035 (1 754 to 2 352)	-21.2% (-34.6% to -5.2%)	627 (468 to 812)	-7.8% (-11.1% to -4.7%)	2 661 (2 321 to 3 031)	-18.4% (-29.6% to -5.5%)
Low-middle SDI	Sudan	62 (49 to 81)	-56.0% (-63.9% to -42.9%)	3 105 (2 514 to 3 892)	-60.5% (-68.6% to -46.1%)	744 (556 to 966)	-3.6% (-7.0% to 1.2%)	3 849 (3 247 to 4 653)	-55.4% (-63.7% to -40.7%)
Low-middle SDI	Swaziland	117 (94 to 142)	-6.4% (-29.0% to 18.4%)	5 532 (4 443 to 6 744)	-4.1% (-27.3% to 21.1%)	644 (474 to 837)	-10.5% (-13.5% to -7.3%)	6 176 (5 081 to 7 375)	-4.8% (-25.6% to 17.5%)
Low-middle SDI	Tajikistan	36 (33 to 40)	-37.4% (-43.4% to -28.9%)	1 878 (1 715 to 2 095)	-38.6% (-44.4% to -31.7%)	1 255 (934 to 1 647)	-1.8% (-6.8% to 5.2%)	3 134 (2 764 to 3 542)	-27.7% (-32.8% to -22.0%)
Low-middle SDI	Timor-Leste	44 (30 to 54)	-55.0% (-69.5% to -45.8%)	1 894 (1 044 to 2 326)	-63.5% (-79.5% to -54.6%)	907 (655 to 1 223)	-8.2% (-15.2% to -0.7%)	2 801 (1 998 to 3 355)	-54.7% (-67.6% to -47.0%)
Low-middle SDI	Vanuatu	85 (64 to 109)	-5.4% (-28.1% to 26.3%)	3 855 (2 813 to 5 085)	-6.7% (-31.6% to 27.3%)	699 (529 to 890)	39.3% (35.6% to 42.8%)	4 555 (3 480 to 5 776)	-1.7% (-24.3% to 29.6%)
Low-middle SDI	Zambia	75 (66 to 84)	-35.7% (-46.4% to -16.2%)	2 628 (2 289 to 3 019)	-41.9% (-53.8% to -11.2%)	761 (566 to 975)	-8.7% (-10.5% to -6.9%)	3 389 (2 980 to 3 830)	-36.8% (-47.7% to -10.5%)
Low-middle SDI	Zimbabwe	103 (89 to 118)	19.6% (-3.9% to 42.8%)	4 144 (3 539 to 4 732)	24.8% (-0.8% to 51.1%)	668 (503 to 852)	18.4% (15.5% to 21.7%)	4 813 (4 167 to 5 449)	23.8% (1.9% to 46.0%)
Low SDI	Afghanistan	103 (86 to 122)	-35.9% (-51.9% to 37.6%)	4 913 (4 067 to 5 876)	-40.9% (-56.9% to 44.4%)	1 995 (1 442 to 2 642)	-42.3% (-48.5% to -34.9%)	6 909 (5 920 to 8 048)	-41.3% (-53.2% to 3.5%)
Low SDI	Bangladesh	42 (37 to 47)	-48.9% (-56.4% to -38.5%)	1 878 (1 628 to 2 153)	-58.3% (-65.4% to -46.2%)	610 (457 to 791)	6.4% (-0.5% to 14.5%)	2 489 (2 209 to 2 810)	-50.9% (-58.3% to -39.0%)
Low SDI	Benin	95 (76 to 118)	-24.5% (-36.8% to -8.9%)	3 565 (2 814 to 4 471)	-33.0% (-45.2% to -18.5%)	659 (488 to 850)	-11.6% (-14.1% to -9.0%)	4 224 (3 450 to 5 160)	-30.4% (-40.9% to -17.6%)
Low SDI	Burkina Faso	94 (83 to 106)	-14.9% (-26.5% to 1.2%)	3 585 (3 043 to 4 244)	-18.2% (-30.6% to -1.9%)	595 (442 to 771)	-5.8% (-8.8% to -3.0%)	4 181 (3 636 to 4 849)	-16.7% (-27.8% to -2.4%)
Low SDI	Burundi	88 (74 to 105)	-34.3% (-45.2% to -19.4%)	3 099 (2 613 to 3 757)	-39.1% (-50.1% to -22.7%)	1 790 (1 298 to 2 346)	87.6% (56.0% to 136.2%)	4 890 (4 156 to 5 817)	-19.1% (-31.5% to -2.1%)
Low SDI	Central African Republic	201 (161 to 235)	27.0% (5.1% to 79.0%)	10 134 (7 937 to 12 026)	31.9% (6.8% to 113.4%)	879 (652 to 1 129)	33.4% (23.1% to 48.2%)	11 013 (8 808 to 12 914)	32.0% (8.6% to 105.1%)
Low SDI	Chad	89 (75 to 108)	-3.9% (-17.2% to 10.7%)	3 577 (3 028 to 4 199)	-12.8% (-27.0% to 3.1%)	776 (584 to 983)	-8.1% (-13.7% to -3.1%)	4 353 (3 770 to 5 057)	-12.0% (-23.8% to 1.0%)
Low SDI	Comoros	67 (56 to 82)	-35.5% (-46.1% to -22.6%)	2 334 (1 905 to 2 948)	-42.6% (-52.8% to -28.9%)	787 (582 to 1 014)	-22.0% (-24.2% to -19.9%)	3 121 (2 631 to 3 805)	-38.5% (-47.3% to -27.2%)
Low SDI	Cote d'Ivoire	89 (77 to 102)	-14.1% (-27.2% to 1.9%)	3 329 (2 849 to 3 867)	-17.7% (-31.0% to -1.2%)	645 (478 to 832)	-4.8% (-6.9% to -2.8%)	3 974 (3 456 to 4 557)	-15.8% (-27.4% to -1.9%)
Low SDI	DR Congo	78 (67 to 91)	-15.4% (-28.5% to 6.5%)	3 624 (3 035 to 4 291)	-21.0% (-35.1% to 6.2%)	770 (585 to 975)	12.4% (6.9% to 20.3%)	4 394 (3 787 to 5 071)	-16.7% (-29.6% to 7.2%)
Low SDI	Eritrea	105 (87 to 125)	-91.6% (-93.0% to -89.9%)	4 025 (3 255 to 4 929)	-94.1% (-95.2% to -92.7%)	1 857 (1 367 to 2 448)	-52.6% (-57.5% to -46.3%)	5 882 (4 940 to 6 952)	-91.9% (-93.1% to -90.4%)
Low SDI	Ethiopia	69 (63 to 77)	-61.0% (-66.7% to -52.6%)	2 378 (2 142 to 2 650)	-68.6% (-73.9% to -60.3%)	951 (709 to 1 211)	-14.4% (-17.5% to -11.0%)	3 328 (2 982 to 3 708)	-61.7% (-67.2% to -53.5%)
Low SDI	Guinea	85 (72 to 100)	-13.4% (-26.8% to 1.0%)	3 238 (2 713 to 3 779)	-28.9% (-41.8% to -12.5%)	647 (481 to 831)	-10.5% (-13.0% to -7.9%)	3 885 (3 331 to 4 489)	-26.4% (-37.7% to -12.0%)
Low SDI	Guinea-Bissau	99 (85 to 116)	-31.7% (-44.2% to -16.9%)	3 796 (3 132 to 4 602)	-37.5% (-50.1% to -20.5%)	632 (471 to 810)	-14.0% (-16.5% to -11.4%)	4 427 (3 753 to 5 218)	-34.9% (-46.7% to -19.9%)
Low SDI	Haiti	108 (90 to 132)	-38.4% (-48.9% to -24.6%)	5 052 (4 206 to 6 110)	-45.1% (-55.0% to -30.4%)	1 433 (1 052 to 1 894)	164.0% (116.7% to 225.8%)	6 485 (5 560 to 7 616)	-33.5% (-44.5% to -18.2%)
Low SDI	Kiribati	63 (52 to 74)	-7.9% (-24.4% to 10.1%)	3 101 (2 532 to 3 653)	-9.5% (-26.7% to 9.4%)	543 (408 to 689)	44.7% (39.8% to 49.5%)	3 645 (3 080 to 4 220)	-4.1% (-20.3% to 13.0%)
Low SDI	Liberia	61 (52 to 73)	-79.6% (-82.6% to -75.8%)	2 173 (1 789 to 2 721)	-86.4% (-88.8% to -82.8%)	825 (620 to 1 053)	-6.9% (-16.5% to 4.6%)	2 997 (2 571 to 3 578)	-82.2% (-84.7% to -78.5%)
Low SDI	Madagascar	65 (54 to 76)	-31.4% (-42.1% to -19.9%)	2 356 (1 954 to 2 831)	-39.9% (-50.4% to -26.2%)	792 (588 to 1 018)	-16.3% (-18.4% to -14.3%)	3 148 (2 686 to 3 677)	-35.3% (-44.1% to -24.2%)
Low SDI	Malawi	67 (59 to 76)	-31.7% (-47.6% to 20.7%)	2 328 (1 998 to 2 712)	-38.6% (-56.3% to 46.1%)	683 (511 to 878)	-7.1% (-9.6% to -4.6%)	3 011 (2 616 to 3 431)	-33.5% (-50.1% to 27.9%)
Low SDI	Mali	77 (64 to 97)	-31.1% (-41.8% to -15.6%)	3 394 (2 845 to 4 117)	-35.7% (-46.6% to -18.6%)	702 (529 to 891)	-2.8% (-8.2% to 4.1%)	4 097 (3 518 to 4 868)	-31.7% (-41.9% to -16.8%)
Low SDI	Mozambique	95 (83 to 109)	-25.7% (-36.8% to -13.1%)	3 477 (2 998 to 4 024)	-35.8% (-45.8% to -24.0%)	893 (673 to 1 134)	-40.4% (-49.4% to -31.6%)	4 370 (3 863 to 4 951)	-36.8% (-44.8% to -27.1%)
Low SDI	Nepal	70 (57 to 85)	-21.0% (-37.7% to 0.4%)	2 499 (1 900 to 3 120)	-36.1% (-51.9% to -10.9%)	675 (510 to 857)	10.5% (6.0% to 15.3%)	3 173 (2 564 to 3 850)	-29.8% (-44.0% to -8.0%)
Low SDI	Niger	71 (58 to 92)	-32.1% (-43.9% to -15.7%)	2 858 (2 295 to 3 583)	-45.2% (-56.5% to -26.3%)	625 (461 to 805)	-13.9% (-16.4% to -11.5%)	3 483 (2 886 to 4 227)	-41.4% (-52.2% to -24.6%)
Low SDI	Papua New Guinea	126 (103 to 150)	-19.4% (-34.2% to -3.1%)	6 181 (5 054 to 7 436)	-21.5% (-36.6% to -5.1%)	623 (471 to 793)	29.7% (25.8% to 33.7%)	6 803 (5 652 to 8 041)	-18.5% (-33.0% to -2.8%)
Low SDI	Rwanda	79 (66 to 96)	-57.9% (-64.6% to -48.8%)	2 694 (2 228 to 3 358)	-65.6% (-71.5% to -56.1%)	1 649 (1 195 to 2 229)	60.5% (33.8% to 102.5%)	4 342 (3 642 to 5 196)	-51.0% (-58.7% to -40.5%)
Low SDI	Senegal	67 (58 to 79)	-18.7% (-30.0% to -5.8%)	2 342 (2 003 to 2 900)	-30.5% (-42.0% to -13.2%)	618 (460 to 798)	-10.2% (-12.4% to -8.0%)	2 960 (2 574 to 3 571)	-27.1% (-36.8% to -12.6%)
Low SDI	Sierra Leone	76 (65 to 89)	-16.0% (-30.1% to 4.7%)	2 978 (2 505 to 3 503)	-30.4% (-43.7% to -9.8%)	781 (586 to 996)	16.2% (7.0% to 30.2%)	3 759 (3 263 to 4 335)	-24.1% (-37.0% to -5.9%)
Low SDI	Solomon Islands	105 (89 to 123)	-17.1% (-33.3% to 2.0%)	4 394 (3 637 to 5 212)	-19.8% (-37.2% to 0.2%)	696 (522 to 889)	29.1% (26.1% to 31.8%)	5 090 (4 279 to 5 893)	-15.4% (-32.0% to 2.9%)
Low SDI	Somalia	150 (120 to 189)	-7.0% (-30.0% to 39.8%)	6 224 (5 056 to 7 791)	-11.8% (-35.8% to 44.7%)	1 141 (848 to 1 462)	5.7% (2.6% to 8.8%)	7 365 (6 143 to 8 961)	-9.5% (-31.0% to 36.7%)
Low SDI	South Sudan	141 (121 to 168)	15.5% (-12.1% to 64.5%)	6 224 (5 439 to 7 291)	25.0% (-6.7% to 94.3%)	1 167 (867 to 1 495)	29.1% (21.1% to 41.3%)	7 392 (6 536 to 8 440)	25.7% (-2.2% to 80.5%)
Low SDI	Tanzania	61 (54 to 69)	-23.7% (-36.8% to 8.7%)	2 258 (1 958 to 2 610)	-25.6% (-41.1% to 29.0%)	739 (554 to 947)	-4.2% (-6.6% to -1.9%)	2 997 (2 639 to 3 382)	-21.3% (-34.5% to 18.8%)
Low SDI	The Gambia	72 (61 to 86)	-7.9% (-22.9% to 10.0%)	2 416 (1 899 to 3 051)	-18.0% (-33.4% to 1.5%)	592 (438 to 764)	-11.2% (-14.6% to -8.2%)	3 008 (2 469 to 3 663)	-16.7% (-29.8% to -1.1%)
Low SDI	Togo	73 (62 to 85)	-14.9% (-29.6% to 2.0%)	2 658 (2 205 to 3 193)	-23.6% (-37.7% to -5.0%)	588 (435 to 756)	-11.0% (-13.6% to -8.1%)	3 246 (2 757 to 3 783)	-21.6% (-34.0% to -6.0%)

SDI Quintile	Location	Deaths (95% UI)		YLLs (95% UI)		YLDs (95% UI)		DALYs (95% UI)	
		2017 age-standardised rates per 100,000	Percentage change in age-standardised rates between 1990 and 2017	2017 age-standardised rates per 100,000	Percentage change in age-standardised rates between 1990 and 2017	2017 age-standardised rates per 100,000	Percentage change in age-standardised rates between 1990 and 2017	2017 age-standardised rates per 100,000	Percentage change in age-standardised rates between 1990 and 2017
Low SDI	Uganda	71<br>(61 to 84)	-18.4%<br>(-33.4% to 0.0%)	2 468<br>(2 095 to 2 997)	-24.8%<br>(-38.5% to -7.4%)	898<br>(680 to 1 134)	-19.3%<br>(-29.2% to -11.2%)	3 366<br>(2 918 to 3 962)	-23.4%<br>(-34.5% to -10.0%)
Low SDI	Yemen	127<br>(111 to 150)	5.3%<br>(-19.2% to 103.1%)	6 511<br>(5 792 to 7 626)	3.4%<br>(-21.8% to 125.9%)	787<br>(587 to 1 011)	-10.3%<br>(-14.6% to -5.6%)	7 298<br>(6 526 to 8 438)	1.8%<br>(-20.7% to 93.8%)
